# MicroMagnify: A Multiplexed Expansion Microscopy Method for Pathogens and Infected Tissues

**DOI:** 10.1002/advs.202302249

**Published:** 2023-09-01

**Authors:** Zhangyu Cheng, Caroline Stefani, Thomas Skillman, Aleksandra Klimas, Aramchan Lee, Emma F. DiBernardo, Karina Mueller Brown, Tatyana Milman, Yuhong Wang, Brendan R. Gallagher, Katherine Lagree, Bhanu P. Jena, Jose S. Pulido, Scott G. Filler, Aaron P. Mitchell, N. Luisa Hiller, Adam Lacy‐Hulbert, Yongxin Zhao

**Affiliations:** ^1^ Department of Biological Sciences Carnegie Mellon University 4400 Fifth Avenue Pittsburgh PA 15213 USA; ^2^ Benaroya Research Institute at Virginia Mason 1201 9th Ave Seattle WA 98101 USA; ^3^ Immersive Science LLC 6835 113TH PL SE Newcastle WA 98056 USA; ^4^ Wills Eye Hospital and Jefferson University Hospital Philadelphia PA 19107 USA; ^5^ Viron Molecular Medicine Institute 201 Washington Street Boston MA 02201 USA; ^6^ Department of Physiology Wayne State University 42 W Warren Ave Detroit MI 48202 USA; ^7^ NanoBioScience Institute Wayne State University 42 W Warren Ave Detroit MI 48202 USA; ^8^ Center for Molecular Medicine & Genetics School of Medicine Wayne State University 42 W Warren Ave Detroit MI 48202 USA; ^9^ Lundquist Institute for Biomedical Innovation at Harbor‐UCLA Medical Center 1124 W Carson St Torrance CA 90502 USA; ^10^ David Geffen School of Medicine at UCLA 10833 Le Conte Ave Los Angeles CA 90095 USA; ^11^ Department of Microbiology University of Georgia 210 S Jackson street Athens GA 30602 USA

**Keywords:** expansion microscopy, infected tissue, microbiology, multiplexing

## Abstract

Super‐resolution optical imaging tools are crucial in microbiology to understand the complex structures and behavior of microorganisms such as bacteria, fungi, and viruses. However, the capabilities of these tools, particularly when it comes to imaging pathogens and infected tissues, remain limited. MicroMagnify (µMagnify) is developed, a nanoscale multiplexed imaging method for pathogens and infected tissues that are derived from an expansion microscopy technique with a universal biomolecular anchor. The combination of heat denaturation and enzyme cocktails essential is found for robust cell wall digestion and expansion of microbial cells and infected tissues without distortion. µMagnify efficiently retains biomolecules suitable for high‐plex fluorescence imaging with nanoscale precision. It demonstrates up to eightfold expansion with µMagnify on a broad range of pathogen‐containing specimens, including bacterial and fungal biofilms, infected culture cells, fungus‐infected mouse tone, and formalin‐fixed paraffin‐embedded human cornea infected by various pathogens. Additionally, an associated virtual reality tool is developed to facilitate the visualization and navigation of complex 3D images generated by this method in an immersive environment allowing collaborative exploration among researchers worldwide. µMagnify is a valuable imaging platform for studying how microbes interact with their host systems and enables the development of new diagnosis strategies against infectious diseases.

## Graphical abstract

MicroMagnify (µMagnify), a new multiplexed expansion microscopy method optimized for pathogens and infected tissues. It enables up to eightfold expansion factor through a robust homogenization process, which is highly compatible with different types of pathogen‐related samples, from planktonic bacteria to infected formalin‐fixed paraffin‐embedded tissue. Due to its biomolecule retention capability, µMagnify allows for multiplexed and multi‐modal nanoscale imaging within the sample.

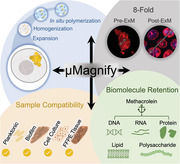

## 1 Introduction

The small size of microbes, varying from 0.15 to 700 µm,^[^ ^1^ ^]^ makes it challenging to investigate the spatial arrangements of biomolecules under conventional optical microscopy, which can only provide ≈ 250 nm resolution. Electron microscopy offers atomic‐level resolution but requires expensive instrument and lab setup, provides little molecular contrast, and involves tedious sample preparation including dehydration and chemical fixation as well as slicing thin sections of the tissue. Super‐resolution imaging techniques evolved remarkably over the last decades to allow new microbiology observations, bringing the resolution down to 10–50 nm. However, these methods come with their own set of challenges such as long training times required for operating sophisticated systems or high costs related to purchasing and maintenance that present barriers, especially for laboratories with limited budgets.^[^ ^2^ ^]^

Expansion microscopy (ExM) is a recently emerged technique that offers remarkable resolving power without the need for expensive, specialized hardware. This approach involves embedding biological samples such as organs, tissues, and cells in superabsorbent polyacrylate gels and anchoring key biomolecules onto the polymer network. After mechanical homogenization of the sample‐gel hybrids, they can be expanded isotropically by immersing them in an aqueous solution to de‐crowd the biomolecules. ExM has been continuously evolving with the improvements including large volume,^[^ ^3^, ^4^ ^]^ expansion factor,^[^ ^5^ ^]^ and multiplexibility.^[^ ^6^, ^7^ ^]^

Despite the impressive advancements in ExM, there has been limited research into microbial imaging applications thus far due to certain drawbacks of current methods: Lim et al. reported that mixed bacterial cultures show incomplete and heterogenous expansion patterns when treated with proteinase K and cell wall digestion.^[^ ^8^ ^]^ This proteinase K treatment also disrupts epitopes and limit multiplexing capability. Published expansion protocols for fungal and bacterial samples are limited to 2–3 targets due to the need to construct fluorescent proteins fusion with their target^[^ ^9^ ^]^ or perform pre‐expansion staining due to proteinase K.^[^ ^10^ ^]^ A recent study showed the possibility of combining heat denaturation with cell wall digestion to expand yeast cells,^[^ ^11^ ^]^ but without distortion measurement. No methods have been demonstrated beyond infected cell cultures,^[^ ^10^, ^12^, ^13^ ^]^ indicating a gap between simple cell infection models and more complex scenarios, such as dense biofilms and infected tissues including those preserved in formalin‐fixed paraffin‐embedded format (FFPE) or stained with hematoxylin and eosin (H&E). More importantly, few systematic characterizations of image distortion between pre‐ and post‐expansion image pairs are done to validate these microbiology‐focused ExM methods,^[^ ^14^, ^15^ ^]^ meaning that development of ExM for nanoscale microbiology imaging remains in its infancy stage overall.

We present microMagnify (µMagnify), a nanoscale imaging platform that enables high‐plex fluorescence imaging of pathogen and infected samples. We found that enzyme‐based methods are insufficient for cell wall homogenization. Our approach combines heat denaturation with a cocktail of cell wall digestive enzymes to efficiently homogenize pathogens with and without tissues. Derived from Magnify,^[^ ^16^ ^]^ µMagnify universally anchors different kinds of biomolecules in the hydrogel. We validated our methods on different representative samples, such as bacterial and fungal supernatants, biofilm, and infected cell cultures/tissue, with a < 4% distortion and up to eightfold expansion factor. Additionally, we demonstrated high‐plex 10‐color 3D nanoscale imaging of DNA, RNA, proteins, lipids, and polysaccharides in microbes through this method. Finally, an immersive visualization tool was designed to allow researchers worldwide to perceive complex datasets from multiple angles, thus sharing knowledge in real‐time.

## 2 Results

### 2.1 µMagnify Expands a Wide Range of Pathogens with Minimal Distortion

Bacterial and fungal pathogens have rigid cell wall envelopes that play an essential role in maintaining cell shape and providing protection from osmotic pressure. The rigid structure of the microbial cell wall poses challenges for isotropic expansion using existing protocols. The common component of the bacterial envelope is a peptidoglycan layer consisting of long strands of glycans covalently crosslinked by stretchable peptides. In addition, Gram‐negative bacteria have an additional outer membrane composed of phospholipids and glycolipids. Gram‐positive bacteria lack this outer membrane but possess thicker peptidoglycan layers for chemical resistance.^[^ ^17^, ^18^ ^]^ Fungal cells on the other hand feature polysaccharides including β−1,3 glucan, β−1,6 glucan, α−1,3 glucans, or chitins with mannoproteins linked to them.^[^ ^19^ ^]^

**Figure 1:**
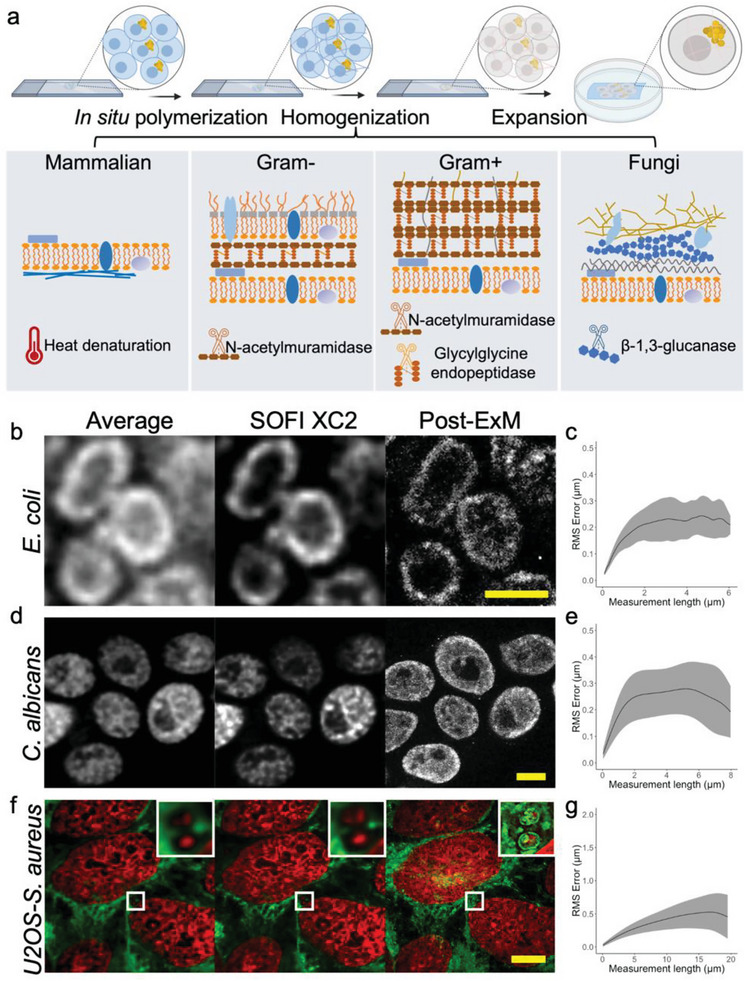
Schematic and validation of µMagnify. a) Brief schematic of µMagnify chemical processing (Figure S1, Supporting Information). Briefly, samples were rehydrated and penetrated for format conversion. Then an appropriate amount of gelling solution was added on top of the specimen for polymer synthesis. The sample‐gel hybrid was mechanically homogenized in denaturing reagents followed by enzyme digestion. The homogenized gel was isotropically expanded in 1:50 diluted PBS or ddH2O for imaging. bg) Validations of µMagnify on representative bacteria, fungi, and bacteria infected cell culture pre‐ and post‐expansion: (b) *E. coli* was stained with CellMask, (d) *Candida albicans* (*C. albicans)* with BODIPY and (f) *Staphylococcus aureus* (*S. aureus)‐*infected *h* *omo sapiens* bone osteosarcoma (U2OS) cell culture with DAPI (red) and BODIPY (green). (b,d,f) Examples of pre‐expansion images taken at 60x and processed with intensity average across 50 frames and XC2 deconvolution SOFI, compared to the post‐expansion images taken at 60x for the same field of view. Post expansion images are Maximum intensity projected over 5–30 frames in z to best match the plane. Biological scales: (b,d), 2 µm; (f), 10 µm. Expansion factor (in 1:50 diluted PBS): (b) 4.97 ± 0.32 (n = 13); (d) 6.06 ± 0.42 (n = 11); (f) 5.79 ± 0.21(n = 16). Right top corner images in (f) are zoomed in images of the white boxed region (length = 2.6 µm). (c,e,g) Root mean square (RMS) length measurement error as a function of measurement length for pre‐expansion SOFI images versus post‐expansion images for (c) *E. coli* (CellMask, n = 13), (e) *C. albicans* (BODIPY, n = 11), and (g) *S. aureus* infected U2OS cell (DAPI, n = 16). Solid line, mean of channel; shaded area, standard error of mean (s.e.m).

We aim to develop an ExM approach optimized for studying biofilm and pathogen‐host interactions in infected specimens. To achieve this, the method should possess certain capabilities: 1) expansion of a diverse range of specimens, including bacteria, fungi, and infected tissues; 2) preservation of diverse biomolecules allowing post‐expansion staining; 3) a straightforward expansion process with resolution similar to traditional super‐resolution imaging methods. We started by employing Magnify – a new ExM method that uses universal anchoring strategies to retain DNA, RNA, proteins, glycolipids, and polysaccharides after expansion.^[^ ^16^ ^]^ However, it soon became apparent that its enzyme‐free homogenization technique was insufficient for expanding microbial cells. We also found that using reported enzyme‐based homogenization^[^ ^8^, ^9^, ^10^, ^12^, ^13^ ^]^ can only expand a small set of specimens, such as *E. coli*, at the cost of losing epitopes post‐expansion. To address the issue of cell wall homogenization, we developed a cocktail of digestive enzymes specifically tailored to various types of cell walls (**Figure** **1**a). Mutanolysin (N‐acetylmuramidase) was used to break down the 1,4‐beta‐linkages between N‐acetylmuramic acid and N‐acetyl‐D‐glucosamine residues in peptidoglycan for both Gram‐positive and Gram‐negative bacteria; lysostaphin (glycylglycine endopeptidase) was added to cleave the crosslinking pentaglycine bridges presented in Gram‐positive bacteria; finally, zymolyase (β−1,3‐glucan laminaripentao‐hydrolase and β−1,3‐glucanase activity) was included for digestion of fungal cell walls. To preserve epitopes for post expansion staining, no proteases such as proteinase K were included in this enzyme mixture.

We observed that incubation in denaturant‐rich solution at 80°C followed by digestion with the enzyme cocktail successfully expanded a variety of bacterial and fungal specimens, including biofilms and infected cell cultures/tissues. We found no substantial morphological difference between pre‐ and post‐expansion images of both bacteria and fungi (Figure 1b,d). We named the new method, microMagnify (µMagnify), where micro‐ refers to microbial cells. We further confirmed a low distortion level (< 4%) achieved by µMagnify on all the specimens we tested, including Gram‐negative and Gram‐positive bacterial as well as fungal samples, comparing between super‐resolution optical fluctuation imaging (SOFI)^[^ ^20^ ^]^ pre‐expansion and confocal microscopy post‐expansion images (Figure 1c,e,f,g). Particularly noteworthy is that, comparing to previous approaches (Note S1, Supporting Information), our process enabled expansion of dense *C. albicans* biofilm with intact septum structure (Figure S2A, Supporting Information), homogeneous expansion factors between host and microbial cells (Figure S2B, Supporting Information), and retention of membrane structure for intracellular pathogens (Figure S2C, Supporting Information) with increased lipid dye accessibility, which could not be stained before expansion (Figure 1f,g).

### 2.2 µMagnify Reveals Intracellular Structure of Microbial Cells with Molecular Contrast

**Figure 2:**
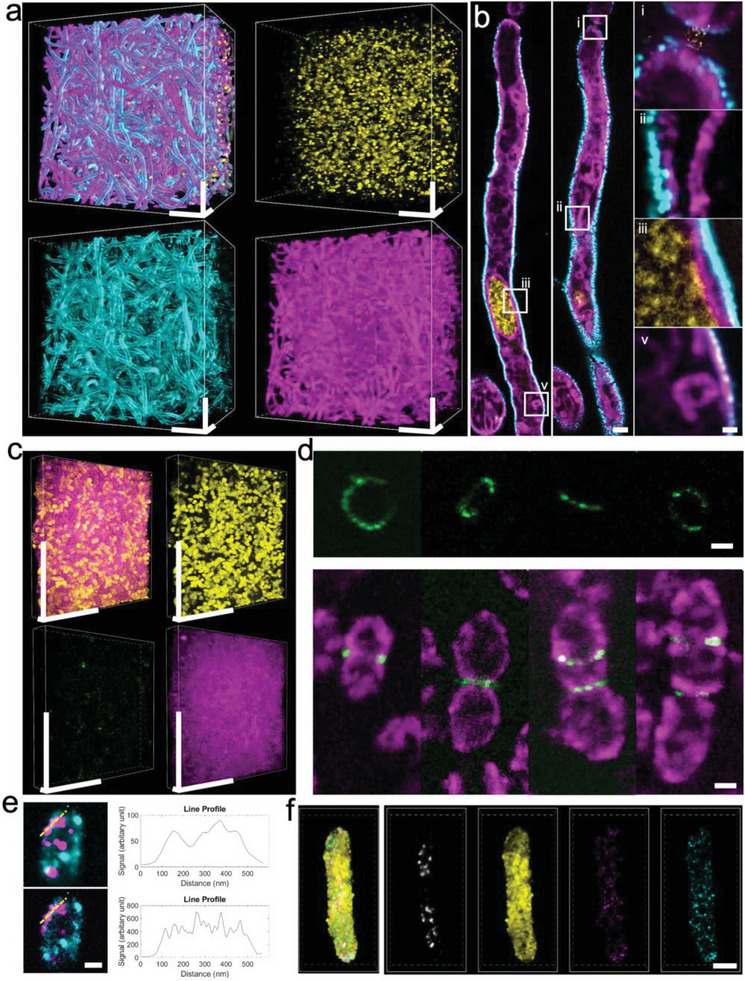
µMagnify works for a diversity of microbial cells, revealing their nanoscale structure and spatial patterns. a) 3D reconstruction of fully expanded *C. albicans* biofilm. The sample was stained with DAPI (yellow), LEL (cyan), and DiI (magenta). Physical scale: 100 µm in x, y, z. b) xy section of hyphae. Zoom‐in views from boxed region showing the hyphae cell junction i) elongated mitochondria beside cell wall ii) details of nuclear membrane, cell membrane and cell wall iii), and lipid body iv). Physical scales: 5 µm (most left and middle columns), 1 µm (most right column). c) 3D reconstruction of *S. pneumoniae* biofilm. The sample was stained with DAPI (yellow), LEL (green), and NHS‐ester (magenta). Physical scales: 100 µm (x, y), 10 µm (z). d) Upper part: examples of ring‐like structures of PG enrichment of dividing *S. pneumonia* at different orientations. PG was stained with LEL. Lower part: examples of *S. pneumonia* constriction by PG at different dividing stages. Cells were stained with LEL (green) and NHS‐ester (magenta). Physical scale: 2 µm. e) Viral particles imaging in E. coli. The sample was stained with wheat germ agglutinin (WGA, cyan) and JCV capsid VP1 antibodies (magenta). The upper part shows spatial distribution of the virus particles in the fully expanded *E. coli* and its intensity profile (upper right) along the yellow dashed line. The lower part shows SRRF processed image from the same ROI and its intensity profile (lower right) along the dashed line. Physical scale: 2 µm. f) Simultaneous protein and RNA imaging of fully expanded *E. coli*, revealing the spatial distribution of mNeon proteins (yellow) and its mRNAs (magenta). The bacteria are also stained with DAPI (gray) and 16s rRNA for control (cyan). Physical scale: 5 µm. All the expansion factors were characterized in Table S1 (Supporting Information).

µMagnify achieved up to eightfold expansion factor (Figure S3 and Table S1, Supporting Information), enabling nanoscale imaging of microbial cell morphology and intracellular organelles with traditional optical microscopes. By clearing opaque specimens and separating densely packed structures, µMagnify provides an effective approach to visualize dense bacterial or fungal biofilms, which have been challenging to image due to the presence of light‐scattering extracellular matrix, light‐absorbing biomolecules, and difficulties identifying individual cells/microcolonies within densely packed structures. This is demonstrated by successfully imaging individual cells in a ≈500 µm thick *C. albicans* biofilm after expansion (**Figure** **2**a). Dil was also used as a lipophilic dye to stain the membranes of individual cells in the biofilm (Figure 2b), highlighting fine structures such as the nucleus envelope and mitochondria. Furthermore, µMagnify successfully imaged densely packed *Streptococcus pneumoniae* (*S. pneumoniae*) biofilm (Figure 2c) along with peptidoglycan (PG) stained by *Lycopersicon esculentum* Lectin (LEL) that revealed PG arrangements at septum rings in individual cells (biological size < 1 µm) at different dividing stages (Figure 2d). The image resolution of µMagnify can be further improved by combining it with other computational super‐resolution techniques without upgrading the microscope; for instance, when combined with super‐resolution radial fluctuations (SRRF) image processing,^[^ ^21^ ^]^ we were able to resolve capsid particles of human polyomavirus viral protein (VP1) overexpressed and self‐assembled inside *E. coli* (Figure 2e) measuring particle sizes ≈ 40 nm, consistent with literature findings.^[^ ^22^ ^]^

µMagnify offers the unique ability to simultaneously image and study multiple types of biomolecules in microbial cells. This technique allows for a diverse range of samples to be studied, including proteins, nucleic acids, lipids, carbohydrates, and more. As demonstrated by examples with *C. albicans* and *S. pneumonia* biofilms (Figure 2a,b), where DNA, carbohydrate, proteins, and lipids were imaged together; *E. coli* (Figure 2f; Figure S4, Supporting Information) which was labeled with mNeon fluorescent protein and its mRNA, and 16s rRNA after expansion; as well as JCV virus expressed in *E. coli* (Figure 2e), which was labeled with anti‐VP1 antibodies after expansion. Therefore, µMagnify enables multi‐modal single‐cell imaging for different types of samples, which is not achievable through existing methods—thus expanding the scope of potential discoveries in microbiology research.

### 2.3 µMagnify Expands Archival Clinical Tissue Specimens with Infection

Formalin fixation is commonly used to generate archival tissue specimens for clinical examination and pathology research. However, it is challenging for traditional ExM methods such as pro‐ExM^[^ ^23^ ^]^ and MAP^[^ ^3^ ^]^ to expand these types of specimens due to heavy formaldehyde‐induced peptidyl crosslinks. Previous work has demonstrated expansion of pathogen‐free formalin‐fixed specimens using aggressive proteinase K digestion but at the cost of losing protein epitopes. Derived from Magnify, µMagnify can expand formalin‐fixed tissue specimens while preserving its protein epitopes after expansion. To maximize preservation, we optimized our protocol by exploring various conditions for homogenization and preincubation (Table S1 and Figure S5ad, Supporting Information). We found that heat denaturation before cell wall digestion was necessary to prevent the formation of cracks in the sample; neither doing heat denaturation after cell wall digestion (Figure S5e,f, Supporting Information) nor performing proteinase K digestion (Figure S5i,j, Supporting Information) provided better preservation than heat denaturation preceding cell wall digestion (Figure S5g,h, Supporting Information).

**Figure 3:**
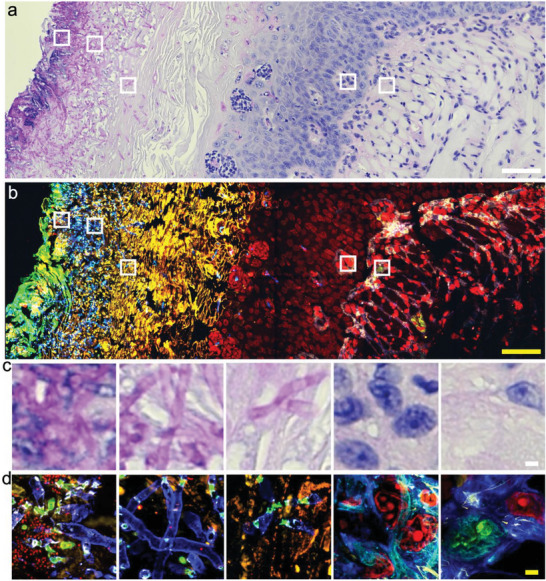
Expansion microscopy imaging of *C*. *albican* *s*‐infected FFPE mouse tongue tissue. a) Bright field pre‐expansion image of *C. albicans‐*infected tissue that was stained with PAS. The cell wall of *C. albicans* were shown in magenta. The nucleus of the tissue is stained in blue. Scale bar: 50 µm. b) Confocal fluorescence image of sample (a) expanded in PBS. The sample was stained with DAPI (red), cellular sugar molecules were labeled differently by WGA (cyan), and LEL (blue). Pan‐proteins were labeled with NHS‐ester (yellow). Biological scale: 50 µm. c) Zoom‐in views of the boxed regions in (a) from left to the right, showing gradient density of C. albicans infection. Scale bar: 2 µm. d) Zoom‐in views of the boxed regions in (b) from left to the right. Biological scale: 2 µm. Expansion factors were characterized in Table S1 (Supporting Information).

We first test µMagnify on formalin‐fixed and periodic acid‐Schiff (PAS) stained mouse tongue infected by *C. albicans*. Bright field microscopic images revealed the presence of *C. albicans* infection, highlighted by magenta staining from the polysaccharide‐enriched cell wall, but lack of clear structural details in high magnification (**Figure** **3**a,c). We used µMagnify to expand the same slide and successfully stained DNA, proteins, and polysaccharides post‐expansion (Figure 3b,d), revealing detailed molecular structures that were not visible in the original PAS stain.

**Figure 4:**
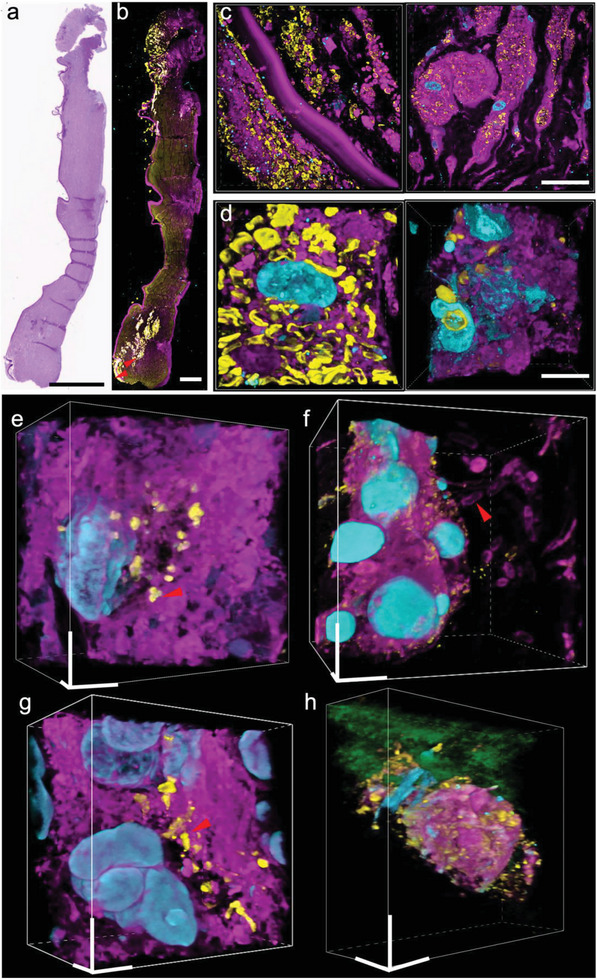
Nanoscale 3D characterization of various pathogen‐infected cornea samples. a) PAS image of FFPE cornea sample of candida keratitis. Scale: 1000 µm. b) µMagnify image of tissue sample cut adjacently to that from (a), taken by 4x objective. Sample were post‐expansion stained with DAPI (cyan), LEL (yellow), and NHS‐ester (magenta). Scale: 1000 µm. c) µMagnify images of pointed region in (b), taken at 10x objective, showing apparent distinction of local *Candida* infection (yellow). Scale: 25 µm. d) Representative images of *C. albicans* interactions with normal (left) and immune (right) cells residing in cornea stroma. eh) Single‐cell level characterizations for various types of eye infections. Samples were stained with DAPI (cyan), WGA (yellow), NHS (magenta), and LEL (green, in h). Scales: 10 µm (x, y, z). (e) Example image of pathogen‐host interactions in *S. epidermidis* (Gram‐positive) keratitis eyeball sample. (f) Example image of extracellular pathogen in *Pseudomonas aeruginosa* (*P. aeruginosa*, Gram‐negative) keratitis cornea sample. (g) Example image of pathogen‐host interaction in atypical mycobacterial (neither Gram‐positive nor Gram‐negative) keratitis cornea sample. (h) Representative images of acanthamoeba located in cornea stroma. Expansion factors were characterized in Table S1 (Supporting Information).

Keratitis is a sight‐threatening disease, primarily caused by infectious agents such as bacteria, viruses, fungi, and protozoa.^[^ ^24^ ^]^ Diagnosing keratitis using histological techniques can be difficult due to the low contrast between pathological features and background tissue, poor resolution of images taken and complexity in identifying different forms of keratitis present within samples examined (Figure S6ae, Supporting Information). Traditional ExM methods with either proteinase K digestion or heat denaturation step do not work for human cornea tissues that consist primarily of dense collagen fibrils embedded in a proteoglycan‐rich matrix; We modified the µMagnify protocol by supplementing collagenase into digestion buffer which proved capable of expanding infected human cornea sections up to 3.6× in PBS or 8× in water (Table S1, Supporting Information). Utilizing µMagnify enabled us to reconstruct 3D images of endothelium cells, epithelium cells and stromal keratocytes residing within the cornea structure itself (Figure S6fh, Supporting Information). Moreover, lectin stains combined with µMagnify enable distinction of different pathogens. For example, LEL strongly stains *C. albicans* cell wall (**Figure** **4**a–d), comparable to H&E stain (Figure S6a, Supporting Information); WGA stain combined with morphological analysis can distinguish Gram‐positive *Staphylococcus epidermidis* (*S*. *epidermidis*) cells (Figure 4e, WGA^+^, round shape), which can be vaguely identified by H&E stain (Figure S6b, Supporting Information), Gram‐negative *pseudomonas* (Figure 4f, WGA^−^, rod shape) that is hard to distinguish the early stage invasion (Figure S6c, Supporting Information), *mycobacteria* (Figure 4g, WGA^+^, rod shape), comparable to Ziehl Neelsen acid fast stain (AFB) image (Figure S6d, Supporting Information), and protozoan infections (Figure 4h, WGA^−^ with much larger size than bacteria), comparable to H&E stain (Figure S6e, Supporting Information). Without the need to use different dyes, µMagnify allows us to accurately differentiate between different forms of keratitis tissues within samples examined with a much higher resolution, thus paving way for potential new diagnostic methods for keratitis.

### 2.4 Signal Unmixing Enabled Multiplexed Imaging using µMagnify

µMagnify's unique ability to retain biomolecules in a hydrogel allows for multiplexed super‐resolution protein imaging in 3D tissues, circumventing the limitation of conventional techniques that struggle to detect more than four targets due to their broad excitation and emission bandwidth. Serial imaging is enabled by this method, allowing different sets of molecules from the same field of view to be imaged sequentially, enabling simultaneous characterization of various players during pathogen‐host interactions. We used computational signal unmixing algorithm^[^ ^25^ ^]^ to deconvolve overlapping signals from different rounds, instead of eliminating existing signals using photoinactivation^[^ ^26^ ^]^ or customized barcoded antibodies.^[^ ^27^ ^]^ We accumulated the signals in raw images, such that true signal of current round is determined by the accumulated raw image subtracted signal from the previous round multiplied by a coefficient (Figure S7a, Supporting Information). Enumeration within a range of possible coefficients will then reveal an optimal coefficient which minimizes the mutual information between the previous round image and true signal (Figure S7b, Supporting Information).

We decided to use the multiplexing and resolution capabilities of µMagnify to visualize intracellular traffic of host proteins and microbes during bacterial infection. We mimicked *S. aureus* infection by treating a human bone osteosarcoma cell line (U2OS) with heat‐killed *S. aureus* in combination with active *S. aureus* alpha‐toxin. We stained the sample with nucleus (DAPI), two distinct groups of carbohydrates (WGA and concanavalin A (ConA)), lipid membrane (DiI), markers of the late endosome/MVB (CD63) and cytoskeleton (Vimentin, α‐tubulin), and pan‐protein (NHS ester conjugated Atto647N). To assess the ability to resolve protein relocalization and colocalization, cells were also stained with two proteins known to interact at endolysosomal membranes, the ubiquitin ligase NEDD4, and LITAF/SIMPLE^[^ ^28^ ^]^ (expressed as a GFP‐tagged fusion and visualized with anti‐GFP antibody).

**Figure 5:**
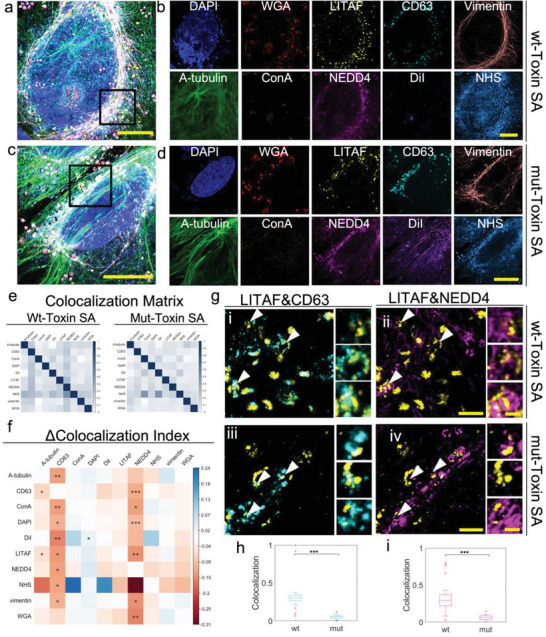
Multiplexing on *S. aureus*‐infected U2OS cell culture allows pairwise study on proteinprotein interactions. a) Ten‐color multiplexed imaging of α‐toxin treated *S. aureus* infected wildtype U2OS cell. The sample was stained with DAPI, WGA, anti‐GFP (targeting LITAF fusion with GFP), anti‐CD63, anti‐Vimentin, anti‐α‐tubulin, ConA, anti‐NEDD4, DiI, and NHS. Biological scale: 10 µm. b) Single color images at different detection channels in (a). Biological scale :10 µm. c) Ten‐color multiplexed imaging of α‐toxin treated *S. aureus* infected LITAF mutant U2OS cell. Sample was stained the same as those in (a). Biological scale: 10 µm. d) Single color images at different detection channels in (c). Biological scale: 10 µm. e) Colocalization matrices calculated for α‐toxin treated *S. aureus* infected wildtype U2OS (left, n = 3) and α‐toxin treated *S. aureus* infected LITAF mutant U2OS cell samples (right, n = 3) among 10 channels. For characterizing colocalization between signal1 and signal2. Colocalization coefficients are calculated as the percentage of the overlapping volume between the signal 1&2 in the volume of signal 1 or signal2. f) Matrix of delta colocalization coefficient between Mut and Wt matrices in (e), indicating the change of pairwise signal colocalization among 10 channels. Asterisks indicated the significant levels through one‐way ANOVA test, ^*^ *p* <0.05, ^**^ *p*<0.01, ^***^ *p*<0.001. g) Representative images of pair analysis for LITAF&CD63 and LITAF&NEDD4 colocalization. The first row showing images i,ii) from boxed region in (a), the second row showing images iii,iv) from boxed region in (c). The first column i,iii) shows composite images of LITAF (yellow) and CD63(cyan). The second column ii,iv) shows composite images of LITAF (yellow) and NEDD4 (magenta). Biological scales: 2 µm. Zoom‐in views of arrow pointed regions (top to bottom) are listed on the right side of each image, delineating the different levels of colocalization between two signals. Biological scales: 500 nm. h,i) Box plot of average colocalization coefficient between Wt (n = 22) and Mut (n = 16) for LITAF&CD63 (h) and LITAF&NEDD4 (i) in *S. aureus*‐containing vacuoles. The middle line in the box shows the median. Bottom and top of each box show the 25th and 75th percentile of the data. Upper and bottom whiskers show the non‐outlier maximum and minimum. Outliers are shown in the red cross. Asterisks indicated the significant differences between Wt and Mut through one‐way ANOVA test, ^***^P <0.001. Expansion factors were characterized in Table S1 (Supporting Information).

All ten stains could be visualized in U2OS cells (**Figure** **5**a–d), and *S. aureus* could be seen at the cell surface or inside endosomes (Figure 5a–d; Figure S8 and Video S1, Supporting Information). The resolution of the image allows for reliable colocalization measurements of every marker simultaneously to investigate the change of proteinprotein colocalization between different samples (Figure 5e,f; Figure S9, Supporting Information). To assess the accuracy of co‐localization measurements, we measured the co‐localization of LITAF with CD63 and NEDD4. It has been previously reported that LITAF localizes to CD63‐positive late endosomes and multivesicular bodies (MVBs) through a mechanism that requires interaction with NEDD4.^[^ ^29^ ^]^ Consistent with these reports, LITAF could be seen colocalized with CD63 and with NEDD4 in endosomal structures surrounding internalized *S. aureus* particles (Figure 5g). Notably, co‐localization was absent for a LITAF mutant that lacks the NEDD4 interaction PPxY motifs (LITAF Y23AY61A) (Figure 5g–i).

### 2.5 Immersive Visualization of Multiplexing Data

**Figure 6:**
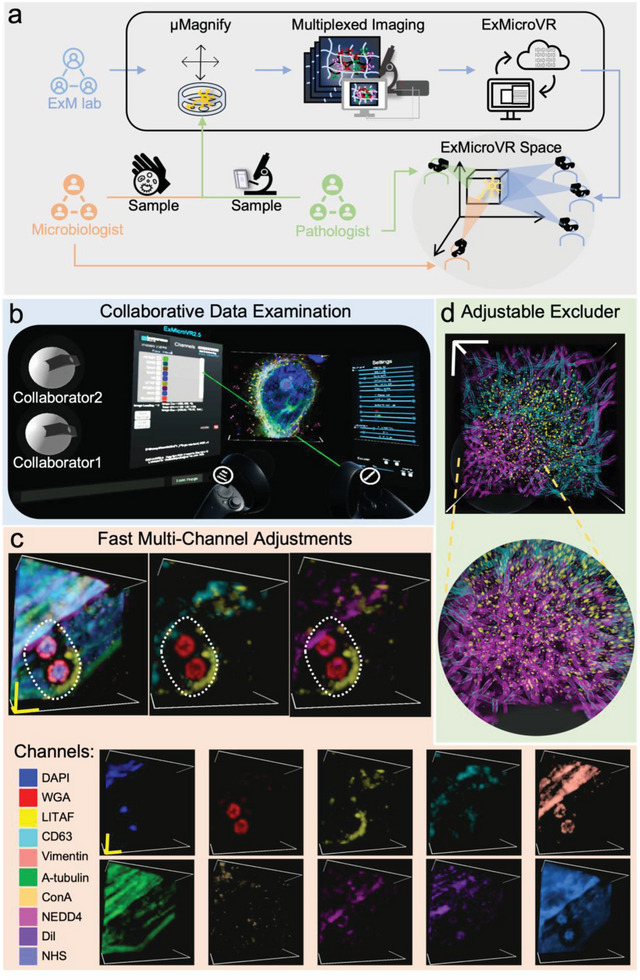
Immersive visualization of multiplexing data and collaboration through ExMicroVR software. a) Workflow of collaboration among ExM, microbiology, and pathology research groups. Microbiologist and pathologist provide samples of infections along with a list of potential biomarkers. ExM lab uses µMagnify to expand the samples and acquire multiplexed images. Images are converted to ExMicroVR‐compatible format followed by data examination and interpretation through immersive visualization and real‐time discussion in ExMicroVR space. b) A representative user view of collaborative data examination through ExMicroVR. 3D multi‐color Image data are presented and adjusted real‐time among joined users. c) Fast multi‐channel data adjustments through ExMicroVR. Example images from mutant cell (Figure 5c) that was stained with DAPI, WGA, anti‐GFP (targeting LITAF fusion with GFP), anti‐CD63, anti‐Vimentin, anti‐Atubulin, ConA, anti‐NEDD4, DiI, and NHS. Biological scales 1 µm in x, y, z. Each channel is easily adjusted and color coded. Composite images can be made to study the interactions between different channels. d) Size‐adjustable excluder applies to inspection of thick biofilm data (Figure 2a), Physical scale: 100 µm.

As µMagnify enables the collection of nanoscale multi‐channel image‐stacks for microbiology and pathology samples, we further developed a virtual reality (VR) application based on ConfocalVR,^[^ ^30^ ^]^ called “ExMicroVR” to provide the researchers an immersive environment for data visualization and exploration that is inaccessible with previous software (**Figure** **6**a). Investigating micro‐scale host‐pathogen interactions is inherently a challenge of interpreting the 3D arrangement of nanoscale protein structures. In a VR environment, selective attention abilities were enhanced from both a behavioral and neural perspective,^[^ ^31^ ^]^ suggesting that ExMicroVR may be a superior visualization approach compared to those typically used in research (e.g., desktop/laptop computers with 2D screens). A representative user view (Figure 6b) as well as the video (Video S1, Supporting Information) demonstrates the ExMicroVR in operation. (Note: 2D videos do not capture the full immersive nature of VR renderings of these complex image‐stacks.). Compared to ConfocalVR, we enhanced the functionalities to accommodate the need to interact with the image stacks, control visualization, analyze the data, and share scientific insights in real‐time among collaborators. The following new features were added: 1) GPU processing load management: ExMicroVR can manage a multi‐channel image‐stack with 10 channels of 10^8^ voxels. The processing load is split between your computer's CPU and GPU, where possible processing is performed on the highly parallel GPU. A comfortable video refresh rate is maintained through the use of channel focus, selective excluders, and adjustable render quality; 2) Multi‐channel adjustments (Figure 6c): selection of channels of interest is done with clicks on a VR “Visualization Control Panel”. Individual or groups of channels can have their parameters adjusted; these settings can also be saved and reloaded; 3) Adjustable Excluder (a form of voxel clipping): improves ROI viewing of inner biological structures packed densely together such as in biofilms (Figure 6d); Excluders can attach to the VR world, image, or virtual “head”. In the “head” mode, areas in front of the viewer are always clear as the user moves around within the image (Video S2, Supporting Information).

We demonstrated ExMicroVR to collect and visualize nanoscale structures of host‐pathogen interactions from data associated with Figure 5 in a collaborative VR environment. With ExMicroVR, we show where the structure of vacuoles around pathogens changes between toxin‐treated *S*. *aureus*‐infected and wild‐type U2OS cells (Figure 6c; Figure S8, Supporting Information). The data generated using our µMagnify platform can provide the basis for further exploration in ExMicroVR, which offers interactive tools to display and analyze the complex 3D architecture of nanoscale structures from many perspectives with enhanced immersive visualization capabilities. This system will likely be useful for understanding how pathogens interact with their hosts at nanoscale resolution to gain insights into pathology and foster novel treatments or strategies against infectious diseases.

## 3 Discussion

We herein described µMagnify, a versatile nanoscale imaging method that enables identification and localization of various biomolecules of fixed pathogen‐infected samples, including proteins, DNA, RNA, lipids, and polysaccharides. We also conducted a valid characterization of the distortion that was not documented in other microbial expansion methods.^[^ ^8^, ^9^, ^10^, ^12^ ^]^ Without the need for dedicated anchoring^[^ ^5^, ^6^ ^]^ or custom linkers to preserve biomolecules,^[^ ^13^, ^32^ ^]^ it is easy to apply µMagnify in various pathogen samples, including Gram‐positive, Gram‐negative bacteria, fungus, virus‐infected cell cultures, and tissues. Note that the enzyme cocktail may not cover all pathogen types due to the unique cell wall components of some microbial species. Customization of the formula might be needed for those exceptional cases. µMagnify is the first expansion microscopy method demonstrated on pathogen‐infected FFPE tissue and H&E slides, enabling potential clinical applications, such as the development of novel diagnoses. In addition, taking advantage of the small size and fast diffusion rate of its monomers makes it possible to expand thick tissue^[^ ^33^ ^]^ and even whole organ.^[^ ^3^ ^]^

The size‐adjustable nature of the hydrogel makes µMagnify a convenient tool for macroscale and microscale inspection of pathogen‐infected samples on conventional imaging system. For microscale biomolecule localization, µMagnify enables approximately eightfold physical expansion at each dimension of the sample to achieve twice resolution enhancement comparing to other microbial expansion methods.^[^ ^8^, ^9^, ^10^, ^11^, ^13^ ^]^ If combined with super‐resolution microscopy, such as Stimulated Emission Depletion Microscopy,^[^ ^34^ ^]^ Structural Illumination Microscopy,^[^ ^35^ ^]^ Single‐molecule Localization Microscopy,^[^ ^36^ ^]^ or post‐imaging processing methods such as SOFI^[^ ^20^ ^]^ and SRRF,^[^ ^21^ ^]^ it could provide even more enhancement of the resolution. For macroscale tissue‐level exploration, imaging the hydrogel in its shrunken state with a high ionic‐concentration buffer allows a speedy construction of tissue atlases using immunostaining or label‐free strategy.^[^ ^37^ ^]^ The combined macroscale and microscale information generated by µMagnify, may reveal previously inaccessible spatial patterns that improve diagnosis of pathogen diseases.^[^ ^38^ ^]^

The multiplexed volumetric 3D data generated by µMagnify and presented by ExMicroVR could lead to systematic analysis with a higher resolution and depth in the field of microbiology and pathology. Furthermore, with retention of diverse biomolecules, µMagnify may be combined with multiplexed protein, DNA and RNA imaging methods, such as immune‐SABER,^[^ ^27^ ^]^ Ab‐oligo cyCIF,^[^ ^39^ ^]^ MERFISH,^[^ ^40^ ^]^ seqFISH+^[^ ^41^ ^]^ enabling characterization of the compositions and interactions between pathogens, and its host in infected cells and tissues with sub‐diffraction limit spatial resolution.

In conclusion, µMagnify is a facile and powerful tool for unearthing pathogen‐infected tissues with nanoscale resolution enhancement. This method could enable more precise diagnosis and novel insights into how infectious diseases progress.

## 4 Experimental Section

### Reagents and Reagent Preparation

The following reagents were used in this study: Paraformaldehyde (PFA, P6148, Sigma–Aldrich), Ethanol (111 000 200, FHARMCO), Xylene (214 736, Sigma–Aldrich), Sodium acrylate (SA, R624, AK Scientific; sc‐236893B, Santa Cruz Biotechnology), N‐dimethylacrylamide (DMAA, 274 135, Sigma–Aldrich), Acrylamide (AA, A8887, Sigma–Aldrich), N,N’‐Methylenebisacrylamide (BIS, M7279, Sigma–Aldrich), Tetramethylethylenediamine (TEMED, T9281, Sigma–Aldrich) 4‐Hydroxy‐2,2,6,6‐tetramethylpiperidine 1‐oxyl (4HT, 176 141, Sigma–Aldrich), Sodium chloride (NaCl, S6191, Sigma–Aldrich), Phosphate buffered saline 10x solution (BP399‐1, Fischer Scientific), Ammonium persulfate (APS, A3678, Sigma–Aldrich), Potassium Persulfate (KPS, 216 224, Sigma–Aldrich), Methacrolein (133 035, Sigma–Aldrich), Ethylenediaminetetraacetic acid (EDTA, 0.5 m, BDH7830‐1, VWR), TritonX‐100 (T8787, Sigma–Aldrich), Tris‐BASE (BP152‐1, Fischer Scientific), Proteinase K (ProK, EO0491, Fischer Scientific), Sodium dodecyl sulfate (SDS, L3771, Sigma–Aldrich), Urea (U5378, Sigma–Aldrich), Glycine (G8898, Sigma–Aldrich), Mutanolysin (M9901, Sigma–Aldrich), Lysotaphin (L7386, Sigma–Aldrich), Zymolyase (E1005, Zymo Research), Ethylene carbonate (EC, E26258, Sigma–Aldrich), Dextran sulfate (50%, S4031, Sigma–Aldrich), 20X SSC buffer (RNase free, AM9763, Fischer Scientific), Tween20 (P1379, Sigma–Aldrich), and Deoxyribonucleic acid, single stranded from salmon testes (Salmon DNA, D7656, Sigma–Aldrich).

SA stock solution was prepared with a final concentration of 50%. ddH2O was added in several times with continuing agitation to ensure complete dissolution (NOTE: ensure enough waiting time for dissolution before volume calibration). Monomer solution was composed of 4% v/v DMAA, 34% SA, 10% AA, 0.02% BIS, 1% NaCl in 1x PBS and stored at 4°C before use. Heat denaturation buffer was composed of 1% SDS, 0.75% Glycine, 8 m Urea, 25 mm EDTA, 500 mm Tris‐BASE in 2× PBS, pH 8.5 at RT. RNA fluorescence in situ hybridization (FISH) buffer was composed of 20% v/v EC, 10% v/v dextran sulfate, 0.1% v/v Tween20, 100 µg mL^−1^ Salmon DNA in 2x SSC. RNA FISH wash buffer 1 was composed of 10% v/v EC in 2x SSC. RNA FISH wash buffer 2 was composed of 0.1% v/v Tween20 in 2x SSC.

### *E. coli* Suspension

DH10B *E. coli* carrying a pBad‐mNeonGreen plasmid (for RNA FISH in Figure 2) were grown overnight shaking at 37°C in LB Broth with 100 mg L^−1^ ampicillin and, if induced, with 100 mg L^−1^ arabinose. DH10B E. coli carrying a pBad‐VP1 plasmid (for capsid VP1 post‐expansion staining in Figure 2) were grown in 3 mL LB Broth with 100 mg L^−1^ ampicillin shaking at 37°C. After 4 h, arabinose was added to a final concentration of 30 mg L^−1^ and the cultures were grown for 18 h at 25°C. To collect the cells pellet, cultures were centrifuged at 4000 g for 5 min. They were then resuspended in 4% paraformaldehyde in PBS 6.8 pH and incubated at room temperature for 30 min. The cells were then washed twice by being pelleted at 1500 g for 5 min and resuspended with PBS.

### *C. albicans* Biofilm

Frozen strain SC5314 was maintained in 15% glycerol frozen stocks at −80°C. Strains were inoculated in liquid YPG at 30°C overnight. Overnight cultures were diluted to an OD600 of 0.2 in 2 ml RPMI media (R5158, Sigma–Aldrich) in 6 well culture plates containing 1.5 cm x 1.5 cm sized medical‐grade silicone squares. After 90 min, the biofilm squares were dipped in sterile PBS to wash unadhered cells and placed in a new 6 well culture plate containing 2 ml of RPMI media. Biofilm cultures (for thick biofilm expansion in Figure 2) were incubated at 37°C with orbital shaking at 60 rpm. After 24 h, biofilms were fixed with ethanol/4% formaldehyde.

### *S. pneumonia* Biofilm

*S. pneumonia* strain D39^[^ ^42^ ^]^ (for biofilm expansion and PG characterization in Figure 2) was grown from frozen stocks by streaking TSA‐II agar plates supplemented with +5% sheep blood. After inoculation into Columbia broth, cultures were incubated at 37°C and 5% CO2 until OD600 reached ≈ 0.05. Cultures (3 mL each) were then added into 6 well chambers that contained coverslips. Biofilm growth was promoted by incubating at 37°C and 5% CO2 for 24 h. Then, the media was carefully aspirated, and dishes were washed twice with PBS. Subsequently, the biofilm samples were fixed with 4% PFA for 20 min. The PFA solution was removed, and the samples were again washed twice with PBS before storage at 4°C until further processing.

### Infected Homo Sapiens Bone Osteosarcoma Cells

U2OS cells were purchased from ATCC (Manassas, VA, USA), and were used at passage < 5. Cells were maintained in McCoy's 5A media, supplemented with 2 mm GlutaMAX, 100 U ml^−1^ penicillin and 100 µg ml^−1^ streptomycin (Gibco), and 10% FBS (Seradigm/VWR). All cells were maintained at 37°C with 5% CO2. LITAF mutant (Y23AY61A) was generated using consecutive quick‐change mutagenesis following vendor protocol on plasmid LITAF‐MycDDK (origene) (cagccactgtctcttcagcggatggaggtgcggatg and gcatgaatcctccttcggcttatacccagccagcgc). GFP‐LITAF constructs were generated by cloning LITAF sequences (WT and mutant), inside pEGFP‐C2 using TOPO cloning. Before transfection, 2×10^5 cells were plated on 6 wells plates and left overnight. Cells were then transfected with 4.5 µl of Fugene 6 (Promega) and 1.5 µg of DNA following manufacturer instructions. The next day, the cells were split at 5×10^4 per well on glass‐bottom wells with removable chambers (Grace Bio‐Lab, Sigma–Aldrich) and treated after 24 h with α‐toxin at 500 ng ml^−1^ and *S. aureus* particles (molecular probes) for 1 h, before fixation in 4% PFA for 15 min.

### *C. albicans* Infected Mouse‐Tongue Sample

BALB/c mouse model of oropharyngeal candidiasis (SC5314) was developed as described in the paper.^[^ ^43^ ^]^ Paraffin‐embedded thin sections of the tongue were obtained and stained with periodic acid‐Schiff stain for histopathology analysis in comparison with expansion images in Figure 3. Details of sample preparations are described in Note S2 (Supporting Information).

### Pathogen‐Infected Eye Sample

Following Wills Eye Hospital Institutional Review Board approval (IRB #2022‐29), 5 pathogen‐infected FFPE corneal tissues (one case each of *Candida parapsilosis*, *P. aeruginosa*, *S. epidermidis*, *Mycobacterium chelonae*, and *Acanthamoeba* keratitis) were retrieved from pathology files. 4 µm thick sections were cut and stained with the following stains: PAS for *Candida* keratitis, H&E for *Staphylococcus* keratitis, Brown Hopps Gram stain (Gram) for *Pseudomonas* keratitis, AFB for *Mycobacterial* keratitis, and H&E for *Acanthamoeba* keratitis. Detailed staining protocols for special microorganisms were provided in Note S3 (Supporting Information) (PAS staining protocol is described below). Appropriate controls were run for each stain. Adjacent cut for each case were processed with µMagnify for pathogen detection in Figure 4.

### Deparaffinization for FFPE Tissue

For FFPE pathogen‐infected tissue, samples were sequentially placed in a series of solutions: 2× xylene, 2× 100% ethanol, 95% ethanol, 70% ethanol, 50% ethanol and (finally) doubly deionized water. All of these steps were performed at room temperature (RT), 3 min each.

### Periodic Acid‐Schiff Staining for FFPE Tissue

Deparaffinize and hydrate tissue slide to water. Oxidize the tissue in 0.5% w/v periodic acid (375 810, Sigma–Aldrich) solution for 5 min. Rinse in distilled water. Place the slide in Schiff reagent (3 952 016, Sigma–Aldrich) for 15 min, as sections become light pink during this step. Rinse slide in lukewarm tap water for 5 min, as sections immediately become dark pink. Counterstain in Mayer's hematoxylin (MHS32, Sigma–Aldrich) for 1 min. Wash in tap water for 5 min. Dehydrate the tissue slide and mount the sample with mounting medium (23‐245691, Fisher Scientific) and coverslips.

### Pre‐Expansion Immunostaining of Pathogen Infected U2OS Cells

After fixation, cell culture was permeabilized for 10 min in 0.5% TritonX‐100 in 1× PBS at RT followed by blocking with SuperBlock Blocking Buffer (37 515, Fisher Scientific) in PBS for 10 min at RT. Samples were then incubated in staining buffer with CellMask green (C37608, Invitrogen, for *E. coli*‐infected U2OS), and BODIPY™ FL DHPE (D3800, Fisher Scientific, for *S. aureus* and *C. albicans*‐infected U2OS) and DAPI (62 248, Fisher Scientific, for *C. albicans*‐infected U2OS) together with 3 h at RT. Samples were then washed at least three times with washing buffer for at least 10 min each at RT.

### In Situ Polymer Synthesis

Immediately prior to gelation, the chemicals 4HT, APS, TEMED, and methacrolein were added to the monomer solution with a final concentration of 0.2%‐0.25% (w/v) APS, 0%‐0.25% (v/v) TEMED, 0.001% 4HT (w/v), and 0.1%‐0.25% (v/v) methacrolein (Table S2, Supporting Information). The solution was vortexed, and the sample were incubated with the gelling solution for 5‐40 min at 4 °C to allow the monomer solution to diffuse into the cell while preventing premature gelation. A gelling chamber was then constructed, consisting of spacers cut from #1.5 cover glass and a glass slide, placed backside down, on top of the cell culture glass that removed from the plate. The samples were incubated overnight in a humidified container at 37 °C to complete gelation.

### Sample Homogenization and Expansion with µMagnify

After gelation, samples were trimmed and incubated in denaturant‐rich buffer (1% w/v SDS, 8 m Urea, 25 mm EDTA, 2× PBS, pH 7.5 at RT). Incubation time depends on sample type: pathogen‐infected cell culture was incubated for 3 h; pathogen‐infected mouse tongue tissues were incubated for 54 h; pathogen‐infected eye samples were incubated for 96 h at 80°C with shaking until the completion of homogenization (i.e., the gelled tissue remains flat without bending or twisting in the solution). Homogenized samples were washed with 1% decaethylene glycol monododecyl ether (C_12_E_10_)/1x PBS for at 60°C twice, at least 15 min per wash, followed by two washes at 37°C, at least 15 min per wash to ensure complete removal of SDS. Samples were further incubated in enzyme solution for cell wall digestion of the pathogens (Table S1, Supporting Information). To improve the expansion factor for thicker samples longer digestion times were required. Finally, gels were placed in ddH2O, 1:50 PBS or 1:50 SSC at RT for 10 min to expand. This step was repeated three to five times until the size of the expanded sample stabilized. Samples could be stored in 1× PBS containing 0.02% sodium azide at 4 °C.

### Post‐Expansion Immunostaining

Samples were incubated with primary antibodies (1:200 dilution) in 2× SSC (300 mm NaCl, 30 mm sodium citrate, pH 7.0)/1% Tween 20 for at least 3 h at RT: rabbit polyclonal anti‐α‐tubulin (11224‐1‐AP, Proteintech), chicken polyclonal anti‐vimentin (ab24525, Abcam), Chicken polyclonal anti‐GFP (ab13970, Abcam), rabbit polyclonal anti‐NEDD4 (PA5‐17463, Invitrogen), mouse monoclonal anti‐human polyoma virus JCV capsid protein VP1 (ab34756,Abcam), mouse monoclonal anti‐CD63 (ab8219, Abcam). Samples were washed four times with PBS for 15 min each at RT, followed by secondary antibodies (1:500 dilution) incubation together with other fluorescent dyes (1:500‐1000 dilution) in PBS for 1–3 h at RT: Donkey Anti‐Mouse IgG (H+L) CF647 (20 177, Biotium), Goat anti‐Chicken IgY (H+L) Alexa Fluor 488 (A11039, Invitrogen), F(ab')2‐Goat anti‐Rabbit IgG (H+L) Alexa Fluor 546 (A11071, Invitrogen), DAPI (62 248, Thermo Scientific), CellMask green (C37608, Invitrogen), BODIPY™ FL DHPE (D3800, Invitrogen), Vybrant DiI (V22885, Invitrogen), *Lycopersicon Esculentum* (Tomato) Lectin DyLight 649 (LEL, DL‐1178, Vector Labs), DyLight 488 (DL‐1174, Vector Labs), Concanavalin A Alexa Fluor 488 (ConA, C11252,Invitrogen), Atto 647N NHS ester (18 373, Sigma), Sulfo‐Cy3 NHS ester (GC17345, GlpBio), Wheat germ agglutinin CF640R (WGA, 29 026, Biotium), WGA Alexa Fluor 488 (W11261, Invitrogen). Note that over 3 h incubation of secondaries will cause potential non‐specific binding.

### µMagnify RNA FISH

Each target gene requires from 20 to 30 probes that consists of gene binding and adaptor regions.^[^ ^44^ ^]^ The gene binding region of RNA FISH probes were designed by LGC bioresearch technologies’ Stellaris RNA FISH Probe Designer (https://www.biosearchtech.com/support/tools/design‐software/stellaris‐probe‐designer). Probe sets were synthesized from IDT on a 96‐well PCR plates. Each gene has a unique fluorophore‐modified imager probes for identification (Table S3, Supporting Information) The RNA FISH protocol^[^ ^45^ ^]^ was modified: the sample was incubated at washing buffer1 at RT for 30 min. Prepare probe mixtures by diluting in hybridization buffer1 at a total probe concentration of 100 nm per gene. Vortex to mix. Probe mixture was added in the gel for 2–3 h incubation at 37 °C. Samples were rinsed in wash buffer1 at 37°C, followed with two washes with wash buffer2 at 37°C and RT. Samples were expanded in 0.02 SSC right before imaging.

### Confocal Imaging

Fluorescence imaging was performed using a Nikon Eclipse Ti2 epifluorescence microscope equipped with a CSU‐W1 spinning disk confocal module and an Andor 4.2 Zyla sCMOS camera. The system was controlled by NIS‐Elements AR 5.21.03 64‐bit software. Images were taken using the following Nikon objectives: CFI Plan Apo Lambda 4× (0.2 NA), CFI Plan Apo Lambda 10× (0.45 NA), CFI Apo LWD Lambda S 20×WI (0.95 NA), CFI Apo LWD Lambda S 40×WI (1.15 NA), CFI Plan Apo Lambda 60×Oil (1.4 NA). DAPI was excited with a 405 nm laser and imaged with a 450/50 emission filter, Alexa Flour 488 was excited with a 488 nm laser and imaged with a 525/40 emission filter. Alexa Fluor 546 was excited with a 561 nm laser and imaged with a 607/36 emission filter. Alexa Fluor 647 was excited with a 640 nm laser and imaged with a 685/40 emission filter. During imaging, the gels were placed in glass‐bottom customed plates with all excess liquid removed. Poly‐L‐lysin coating was recommended recycled imaging plates.

### SOFI Image Preparation

Before evaluating expansion distortion (Figure 1b–g), SOFI method was applied to improve the image resolution of the pre‐expansion image. This method takes higher order statistical analysis of stochastic temporal fluorescence fluctuations of emitters recorded in a sequence of images. By taking the *n*th‐order cumulant of the original pixel time series, the fluorescence signal of the emitters within the pixel was preserved due to higher correlation values across time series, leading to an increased resolution. SOFI images were taken with CFI Plan Apochromat VC 60×C WI (1.2 NA). Each SOFI image consisted of 50–100 frames per z‐plane with 100 ms exposure time per frame. SOFI images were processed using custom MATLAB code. Images were corrected for drift and intensity, cropped, and deconvolved (Lucy‐Richardson method) after 3D cross‐correlation SOFI.

### Measurement of the Expansion Factor

For samples that have pre‐ and post‐expansion image pairs, expansion factors were determined by scale invariant feature transform (SIFT) key points distance (details in next section). For *E. coli* suspensions, expansion factors were estimated by average particle areas. Bacterial particle areas were determined using the Analyze Particles tool in FIJI/ImageJ after image thresholding and binarization. To calculate the linear expansion factor, the square root of the ratio of the average post‐expansion to average pre‐expansion particle area was calculated. For *S. pneumonia* biofilm and *C. albicans* biofilm, expansion factors were estimated by average cell width. Cell widths were determined by manually measuring line length in Analyze FIJI/ImageJ. The ratio of average length pre‐ and post‐expansion were calculated by randomly sampling the images, as estimated liner expansion factor. For FFPE eye tissue, the expansion factor was calculated by measuring the tissue size, pre‐ and post‐expansion. Expansion factors for each type of sample were used to normalize post‐expansion images into biological scale. Normalization process also nulls out the small (<10%) natural sample‐to‐sample variability of the expansion process, as the “biological” length units were obtained out of the total pool of field of views of the same type of samples.

### Measurement Error Quantification

Distortion vector field was generated to calculate the root‐mean‐square (RMS) error as previously described.^[^ ^46^ ^]^ Briefly, for the same field of view, pre‐expansion SOFI images were taken at a single z‐plane at 60× magnification and post‐expansion images were obtained with multiple z‐planes at 60× magnification. To find the best matching post‐expansion z‐planes, SIFT key points were generated for all possible combinations of pairs of the pre‐expansion images and post‐expansion z projections. Different expansion factors and imaging conditions might lead to one post‐expansion z projection from 5 to 30 z planes that corresponds to the pre‐expansion z. SIFT key points were generated using the VLFeat open‐source library and filtered by random sample consensus (RANSAC) using a geometric model that only permits rotation, translation, and uniform scaling. The pair of pre‐expansion and post‐expansion images with the most SIFT key points were then used for image registration, uniform scaling, calculation of expansion factors and distortion vector fields. Manual rigid image alignment by FIJI/ImageJ was applied, when the SIFT algorithm fail to recognize appropriate number of SIFT key points. By subtracting the resulting vectors at any two points, distance measurement errors could easily be sampled, and the RMS error for such measurements was plotted as a function of measurement length from at least three technical replicates.

### Multiplexed Fluorescence Imaging for U2OS Cells

Our gel‐sample hybrid was subjected to multiple rounds of staining without stripping off the previous‐round staining, for the maximum preservation of the targeting biomolecules. This approach also speeds up the staining process for multiplexing. Wildtype and mutant U2OS cells were gelled and homogenized, followed by three cycles of post‐expansion immunostaining and confocal imaging. Gel was expanded in 1:50 PBS before each round of imaging. For accessing the same ROI at every round of imaging. A stitched map image was first captured at 4x magnification with multipoint mode. According to the map, ROIs for each individual cell was found and imaged at 60x magnification with DAPI as the reference channel for image registration.

### Multiplexed Fluorescence Image Processing

After acquisition of three rounds of images for a batch of ROIs, signals from the spectrum‐overlapping antibodies in each channel accumulated after each round of staining and imaging, which requires signal unmixing to specify signals that belong to each biological target. First, images for each ROI were aligned by SIFT rigid registration by rotation, translation, and uniform scaling as mentioned above. For minor local misalignment, images were then subjected to diffeomorphic demons' registration.^[^ ^47^ ^]^ The displacement field was calculated between DAPI channels from the two consecutive rounds to transform images from other three channels. Registered images were then processed with signal unmixing to parcel out signals from individual markers. We customized a signal unmixing algorithm. The signals between the two consecutive rounds (ImageR1 and ImageR2) follows this relationship: Image R_i_ = α^*^Image_i‐1_+’True'Ri (Equation 1). If the unmixing was done, the mutual information between ImageR1 and subtracted ImageR2 should be minimum, considering the stains were targeting two unique biological molecules (Equation 2). Therefore, there exists an optimal α factor that makes the mutual information between ImageR1 and ImageR2‐ α^*^ImageR1 minimum, we could use this α_opt_ to subtract ImageR1 from ImageR2 to get the pure signal of the second staining (Equation 3).

(1) $${\mathrm{Image}}_{i}={\mathrm{ImageR}}_{i}+\alpha^{*}\mathrm{Imag}e_{i-1}$$

(2) $$\alpha_{\mathrm{opt}}=\underset{\alpha }{\arg\min I}\left({\mathrm{Image}}_{i-1};{\mathrm{Image}}_{i}-\alpha^{*}\mathrm{Imag}e_{i-1}\right)$$

(3) $${\mathrm{ImageR}}_{i}=\mathrm{Imag}e_{i}-\alpha_{{\mathrm{opt}}^{*}}\mathrm{Imag}e_{i-1}$$

### Colocalization Analysis

To investigate the interactions among ten channels within each cell, we conducted a colocalization analysis. After background subtraction, registered 10‐color image stacks were converted into a 4D matrix with the fourth dimension representing different channels. The colocalization of each possible two‐channel combinations out of the ten channels were quantified to generate a 10×10 colocalization matrix. First, each channel data, as a 3D matrix was binarized by adaptive local thresholding with adjusted sensitivity (according to the SNR of each channel). For each colocalization test, particle1 from matrix1, particle2 from matrix2 and colocalized particles between two matrices were defined, labeled, and filtered (according to the SNR of each channel) by 26‐connectivity. The colocalization index were calculated as the volume percentage of overlapping particles in particle1 and particle2. The colocalization matrix was generated by averaging the colocalization index across different ROIs for wildtype and mutant sample. Then a delta‐colocalization matrix was calculated to show the change of colocalization between each two channels that was caused by the mutation in the cell. Particularly, we also interested in the colocalization between LITAF and NEDD4/CD63 in SA‐containing vacuoles. We use FIJI ImageJ to manually crop those vacuoles from wildtype and mutant cells and run the colocalization test for LITAF&NEDD4 and LITAF&CD63 channels.

### Statistical Analysis

All experiments were carried out at least three times independently, unless otherwise noted in the figure legends. All data were expressed as standard error of the mean (s.e.m), unless otherwise specified. The following sample sizes were used: Figure 1c: *E. coli* CellMask stain (n = 13 technical replicates from 1 culture). Figure 1e: *C. albicans* BODIPY stain (n = 11 technical replicates from 1 biofilm culture). Figure 1g: *S. aureus* infected U2OS cell (n = 16 technical replicates from 1 culture). Figure 5e: Colocalization matrices for α‐toxin treated *S. aureus* infected wildtype U2OS and α‐toxin treated *S. aureus* infected LITAF mutant U2OS cells, both (n = 3 technical replicates from 1 culture). Figure 5f: one‐way ANOVA test showed statistical difference for the colocalization coefficient between wt and mut infected cells. P values of ANOVA test for each interclass were summarized in Table S4 (Supporting Information). For statistical significance, ^*^ *p* <0.05, ^**^ *p*<0.01, ^***^ *p*<0.001. Figure 5h,i: for LITAF&CD63 and LITAF&NEDD4 colocalization in *S. aureus*‐containing vacuoles. Wt (n = 22 technical replicates from 1 gel), Mut (n = 16 technical replicates from 1 gel). One‐way ANOVA test showed statistical difference for the colocalization coefficient between wt and mut infected cells. F value for LITAF&CD63 colocalization was 35.08 df1 = 1, df2 = 36, *p*<0.001; F value for LITAF&NEDD4 was 26.3, df1 = 1, df2 = 36, p = 0.0001. For statistical significance, ^*^ *p* <0.05, ^**^ *p*<0.01, ^***^ *p*<0.001.

## Conflict of Interest

The authors declare the following competing financial interest(s): Y.Z., A.K., and F.F. are inventors on several inventions related to ExM methods.

## Author Contributions

Z.C. and Y.Z. all contributed key ideas, designed, and executed experiments, and analyzed data for all samples. Z.C. and A.K. developed gel chemistry for infected samples. A.K. and B.R.G. advised on SOFI experiments for infected cell cultures. C.S. designed infected cell culture studies and prepared cell culture samples for µMagnify. C.S. and T.S. developed the virtual reality visualization tool for µMagnify. A.L. assisted with the gelation, staining and imaging for *C. albicans* infected mouse tongue and infected cornea samples. E.D. prepared the *E. coli* suspension samples. K.M.B. and N.L.H. prepared the *S. pneumoniae* sample. A.M. prepared the *C. albicans* biofilm. S.F. prepared *C. albicans*‐infected mice samples. T.M. and J.P. prepared and annotated the infected eye samples. All authors contributed to the writing of the manuscript. Y.Z. supervised the project.

## Supporting information

Supporting Information

Supplemental Video 1

Supplemental Video 2

## Data Availability

Download site for the free ExMicroVR tool and shared repository for μMaginfy generated image stacks can be found at https://www.immsci.com/home/exmicrovr/. Other data are available upon reasonable request to the paper's corresponding author.
