# Supplementary material for: MicroMagnify: A Multiplexed Expansion Microscopy Method for Pathogens and Infected Tissues
Source: Adv Sci (Weinh). 2023 Sep 1;10(30):2302249. doi: 10.1002/advs.202302249 (PMC10602566; doi:10.1002/advs.202302249)
Supplement: Supplementary file 1 — Supporting Information [file ADVS-10-2302249-s003.pdf]

ADVANCED SCIENCE
Open Access

Supporting Information

for *Adv. Sci.*, DOI 10.1002/advs.202302249

MicroMagnify: A Multiplexed Expansion Microscopy Method for Pathogens and Infected Tissues

*Zhangyu Cheng, Caroline Stefani, Thomas Skillman, Aleksandra Klimas, Aramchan Lee, Emma F. DiBernardo, Karina Mueller Brown, Tatyana Milman, Yuhong Wang, Brendan R. Gallagher, Katherine Lagree, Bhanu P. Jena, Jose S. Pulido, Scott G. Filler, Aaron P. Mitchell, N. Luisa Hiller, Adam Lacy-Hulbert and Yongxin Zhao**

MicroMagnify: a multiplexed expansion microscopy method for pathogens and infected tissues

Zhangyu Cheng[1±], Caroline Stefani[2], Thomas Skillman[3], Aleksandra Klimas[1], Aramchan Lee[1], Emma F. DiBernardo[1], Karina Mueller Brown[1], Tatyana Milman[4], Yuhong, Wang[1], Brendan R. Gallagher[1], Katherine Lagree[1], Bhanu P. Jena[5,6,7,8], Jose Pulido[4], Scott G. Filler[9,10], Aaron P. Mitchell[1,11], N. Luisa Hiller[1], Adam Lacy-Hulbert[2], Yongxin Zhao[1]*

[1]Department of Biological Sciences, Carnegie Mellon University, Pittsburgh, PA, USA.

[2]Benaroya Research Institute at Virginia Mason, Seattle WA, USA.

[3]Immersive Science LLC, Newcastle WA, USA.

[4]Wills Eye Hospital and Jefferson University Hospital, Philadelphia, PA, USA.

[5]Viron Molecular Medicine Institute, One Boston Place, 26th Floor, Boston, MA 02108 USA.
[6]Department of Physiology, Wayne State University, Detroit, MI 48201, USA.

[7]NanoBioScience Institute, Wayne State University, Detroit, MI 48201, USA.

[8]Center for Molecular Medicine & Genetics, School of Medicine, Wayne State University, Detroit, MI 48201, USA.

[9]Lundquist Institute for Biomedical Innovation at Harbor-UCLA Medical Center, Torrance, California, USA.

[10]David Geffen School of Medicine at UCLA, Torrance, California, USA.

[11]Department of Microbiology, University of Georgia, Athens, Georgia, USA.

[*]Correspondence to yongxinz@andrew.cmu.edu

This file includes:

Supplementary Notes 1-3
Supplementary Figures 1-9
Supplementary Tables 1-4
Supplementary Videos 1-2
Reference

Supplementary Notes

Supplementary Note 1: Comparison of distortion among μMagnify and other Published expansion methods.

For 4% PFA fixed *Candida albicans* (*C. albicans*) biofilm sample, a staining buffer containing DAPI and LEL in PBST (0.1% Tween20) was applied for pre-expansion imaging. Samples were washed three times before imaging with Nikon CFI Plan Apo VC 60× C WI (1.2 NA). Samples were then subjected to μMagnify, Chen2021[1] and Korovesi2022[2]. Briefly, for Chen2021, C. albicans biofilm were resuspending in 1.2M D-Sorbitol in 0.1M KH2PO4. 100 μL of digestion solution containing 10U/mL Lyticase 1M sorbitol, and 50mM Tris buffer was added in the mixture. Incubated for 30min at 30◦C with gentle shaking. Before polymerization, the sample was incubated with 0.25% glutaraldehyde (GA) for 10min at room temperature and washed several times in PBS. A monomer solution (8.625% sodium acrylate (w/w), 2M NaCl, 2.5% acrylamide (w/w), 0.15% N, N,-methylenbisacrylamide (w/w) in 1x PBS) was added with 0.2% (w/w) tetramethylethylenediamine (TEMED) 1:50 with 10% stock solution 0.2% (w/w) ammonium persulfate 1:50 with 10% stock solution. Sample was incubated with monomer solution at RT for 5 mins. After 2 hrs polymerization at 37℃, the gel was digested in 8 U/mL proteinase K in digestion buffer (50mM Tris pH 8.0, 0.5% Triton X-100, 1mM EDTA, and 0.8M guanidine HCl) for isotopic expansion, for 1hr at 37℃. Digested gels were completely submerged in fresh ddH20 every 0.5 h 3–5 times unless the gel size did not increase. For Korevesi2022, sample was resuspended in 6 mL of sorbitol buffer (1.2 M sorbitol solution in 0.1 M KH2PO4). Add 0.3 μL of zymolyase (5 U/μL) to 200 μL of fixed cells solution, incubate at 30 °C for 10 minutes. 1 mL of 0.1 mg/mL acryloyl X-SE solution in PBS was added to the sample and allow incubation for 12 hours (overnight) at RT. Cooling the reagents (monomer solution, 99% TEMED, and 50% APS) and the gelation chamber slides on ice for at least 10 minutes prior to gelation. Monomer solution contains: 19 g/100 mL SA, 10 g/100 mL AA, 0.1 g/100 mL Bis in 1x PBS. 2.5 uL TEMED stock solution was added to the monomer solution (493ul), followed by adding 5 uL APS stock solution. The mixture was vortexed thoroughly. The slides were kept for 5 more minutes on ice then incubated for 1 hour at 37 °C in a humidified chamber. Coverslip was carefully removed from the slide and the polymerized gel was transferred to a tube. 2 mL of denaturation buffer was added to incubate the gel for 15 minutes at RT. After incubation at 95 °C for 90 minutes, the gel was poured from the Eppendorf tube to a big clean plastic plate to remove excess denaturation buffer. ddH2O was added to Petri dish until the gel is completely submerged. Change ddH2O 2x after 30 minutes and expand the gel in ddH2O overnight at RT. For *C. albicans*-infected U2OS cells, Chen2021 method was applied the similar way. The staining (Dil in PBST buffer), digestion and GA anchoring were all applied to the cell culture coverslip in the petri dish.

For *S. aureus*-infected U2OS cells, a staining buffer containing DAPI and Dil in PBST (0.1% Tween20) was applied for pre-expansion imaging at RT for 1 hr. For Götz2020, samples were incubated overnight at 37°C in either PBS containing 0.02–2 mg mL−1 lysozyme (ThermoFisher Scientific, Waltham, MA) to digest bacterial cell walls. Sample was treated for 10 min with 0.25% GA at RT and gelated after three washing steps. 1ml of the monomer solution containing 0.267 g DMAA (Sigma, 274135) and 0.064 g sodium acrylate (Sigma, 408220) dissolved in 0.57 g ddH2O was degassed for 45 min on ice with nitrogen followed by the addition of 100 μl KPS (0.036 g/ml, Sigma, 379824). After another 15 min of degassing and the addition of 4 μl TEMED per ml monomer solution, gelation was performed for 30 min at RT followed by an incubation of 1.5 h at 37 °C. Hereafter the samples were digested for 3 hrs in digestion buffer

(50mM Tris pH 8.0, 1mM EDTA (Sigma, ED2P), 0.5% Triton X-100 (Thermo Fisher, 28314) and 0.8M guanidine HCl (Sigma, 50933)), supplied with 8 U/ml protease K (Thermo Fisher, AM2548). For Kunz2021, the culture cell coverslip was turned upside down on a drop of the monomer solution (8.625% sodium acrylate, 2.5% acrylamide, 0.15% N, N′-methylene bisacrylamide, 2 M NaCl in 1x PBS) containing freshly added 0.2% ammonium persulfate and tetramethyl ethylene diamine for polymerization. The gel was allowed to polymerize for 90 min at room temperature. The polymerized gel was then removed from the glass slides with tweezers and transferred to digestion buffer (50 mM Tris pH 8.0, 1 mM EDTA, 0.5% Triton X-100 and 0.8 M guanidine HCl, containing 5 mg/mL lysozyme and 50 µg/mL. After 20 min, 8 U/ml protease K (Sigma, P4850) was added for another 30 min. Afterwards, gels were washed and expanded in excess of ddH2O.

***Supplementary Note 2: Candida albicans* infected mouse-tongue sample preparation.**
Three days before infection, inoculate a colony of the C. albicans strain SC5314 into 10 ml of YPD broth and incubate it overnight at 30 °C with shaking at 200 r.p.m. The next day, transfer 100 µl of the overnight culture to 10 ml of fresh YPD broth and incubate it overnight at the same condition. Repeat this step one more time. The day before the infection, weigh the BALB/c mice (18–25 g; Taconic Farms, cat. no. Balb-M). Use their average weight to calculate the dose of cortisone acetate (Sigma-Aldrich, cat. no. C3130), which should be administered at a concentration of 225 mg/kg in a total volume of 0.2 ml. Use a 1-ml syringe with a 5/8-inch, 25-G needle to inject the animal with 0.2 ml of cortisone acetate in sterile PBS containing 0.05% (v/v) Tween 80 subcutaneously in the dorsum of the neck. Place the isothermal pads in a 60 °C water bath overnight. On the day of infection, reduce the water bath temperature to 37 º C before use to avoid overheating the mice. Add 1 ml of the YPD overnight culture to 9 ml of sterile PBS. Centrifuge at 1,000g for 5 min. Decant the supernatant, resuspend the pellet in 10 ml of sterile PBS. Repeat the centrifuge step and resuspend the pellet in 10 ml of sterile HBSS (Sigma, Cat. No. H9269). Dilute the aliquot to make up a suspension of $1x10^6$/ml organisms in 5 ml of sterile HBSS. Warm the C. albicans suspension to 30 °C in a dry block and place the calcium alginate swabs in the suspension for 5 min before they are used to inoculate the mice. Remove the isothermal pads from water bath and place two of them on the stainless-steel pan covered with a paper towel. Inject each mouse intraperitoneally with an anesthetic mixture consisting of 10 mg/ml ketamine (Western Medical Supplies, Cat. No. 4165) and 1 mg/ml xylazine (Western Medical Supplies, Cat. No. 5533) in sterile PBS, administering 0.1 ml per 10 grams of body weight. After the mouse is anesthetized in 20-30 min, place the mouse in the supine position on the isothermal pad with a saturated calcium alginate swab placed sublingually for 75 min. At the first and third day after infection, inject the animal with 0.2 ml of cortisone acetate subcutaneously in the dorsum of the neck with the same dose as before. Five days after infection, administer the anesthetic mixture. Once the mouse is anesthetized, euthanize it by cervical dislocation. Excise the tongue and attached oral tissues by dissecting scissors and forceps and place them on a petri dish. Fix the tongue in zinc-buffered formalin for 4 hr at RT. Fixed tissue can be stored in 80% v/v ethanol before processing for histopathology. For paraffinization, the sample was dehydrated in a series of ethanol solution: 80% v/v, 95% v/v, 95% v/v, 100% v/v,100% v/v, 100%v/v each for 1hr. Sample was washed twice with Citrosolv™ (Fisher Scientific, Cat. No. 04355121), 30 min each time. Place the sample in 60 °C pre-heated paraffin, repeat this step twice more. Immediately prior to embedding, spray wax mold with Mold Grease (IMS, Cat. No. 105998) and coat the bottom layer of the mold with melted paraffin. Place the

sample on top of the base wax. Partially cover the sample with more paraffin. Place pathology cassette on top of the mold and completely fill the mold. Place mold with sample on cooling embedding station until the sample solidify in 10-15 min. The embedded sample was removed from the mold and subjected to sectioning at a thickness of 5 μm.

Supplementary Note 3: Special staining protocols for microorganism-infected eye sample.
Hematoxylin-Eosin Stain (H&E): H&E stain was performed on an automated Leica XL stainer, (model ST5010, Serial number 44571, Leica Biosystems) with the following protocol: xylene (4 minutes), 100% ethanol (2 minutes), 95% ethanol (1 minute), 70% ethanol (1 minute), distilled water (1 minute), hematoxylin (5 minutes), distilled water (1 minute), acid alcohol (1 second), distilled water (1 minute), bluing reagent (5 seconds), distilled water (1 minute), 95% ethanol (5 minutes), eosin (5 minutes), 95% ethanol (2 minutes), 100% ethanol (2 minutes), xylene (4 minutes).
Brown Hopps Gram Stain: Stain with crystal Violet (2 minutes), rinse with distilled water, mordant in Gram's iodine solution (5 minutes), rinse with distilled water, differentiate in cellosolve (5-10 seconds), rinse with distilled water, stain with Basic Fuscin (5 minutes), rinse with distilled water, stain with Gallego's differentiating solution (5 minutes), rinse with distilled water, tartrazine solution (3 seconds), cellosolve (3 changes, 10 dips each), xylene (3 changes, 10 dips each).
Ziehl Neelsen acid fast stain (AFB): Stain with Carbol Fuchsin Ziehl Neelsen (30 min), wash in running water, Acid alcohol 1% until sections are pale pink, wash in running water (8 minutes), counter stain in Methylene Blue Working, wash with tap water, rinse with distilled water, dehydrate in 95% alcohol, absolute alcohol, and clear in Xylene (2 changes each).

Supplementary Figures

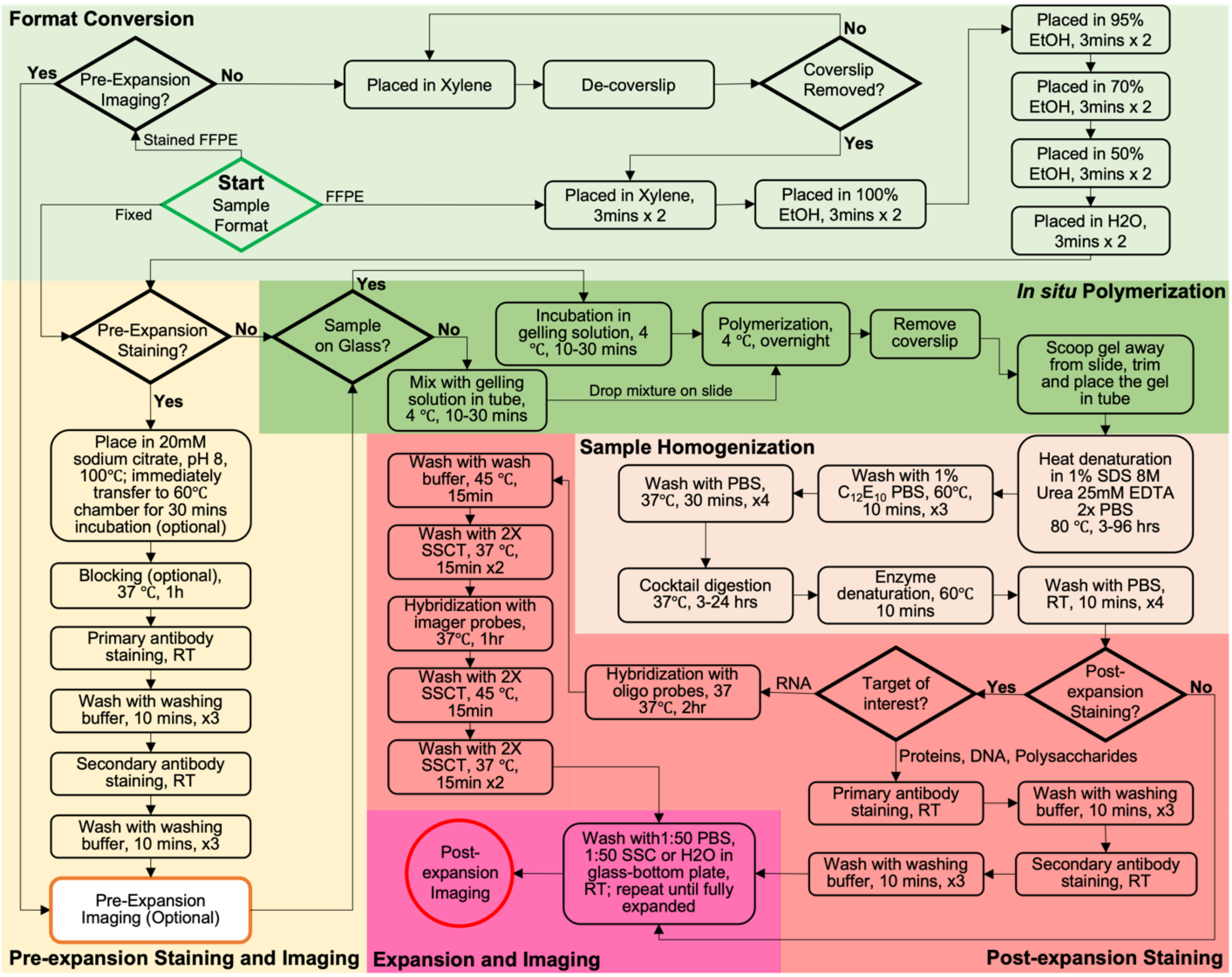

Figure S1: The full workflow for μMagnify.

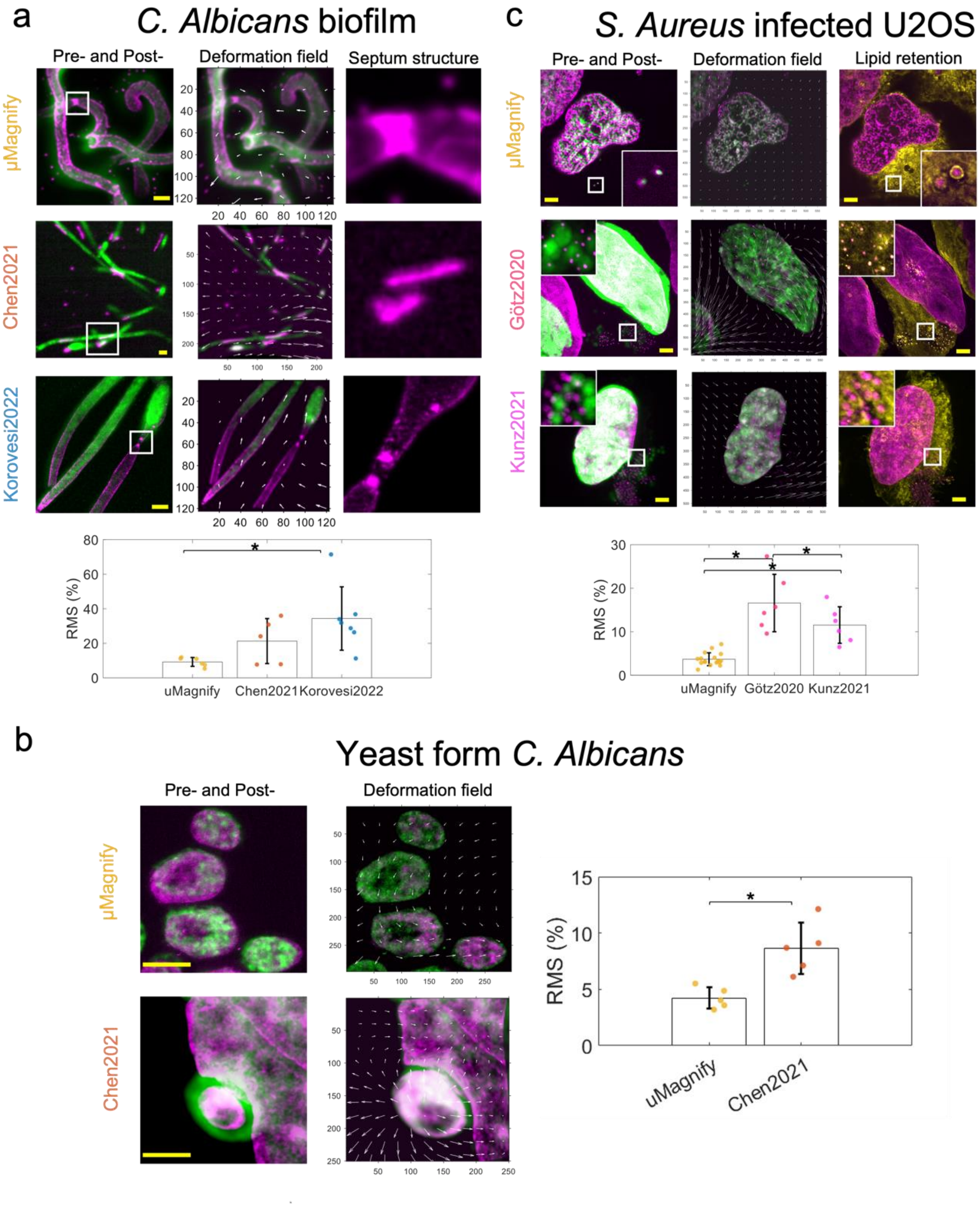

Figure S2: Comparison of distortion among µMagnify and other published methods for various pathogen expansion. (a) *Candida albicans* (*C. albicans*) biofilm was fixed with 4% PFA and stained with LEL (green and magenta for pre- and post- images). Samples were subjected to different expansion methods: µMagnify, Chen2021[1] and Korovesi2022[2]. Deformation field between pre-expansion images (green) taken by Nikon CFI Plan Apo VC 60× C WI (1.2 NA) and post-expansion images (magenta) imaged with the same microscopy setting were overlayed on the registered images in the middle column. Biological scales 2 µm. Right column shows zoom-in views of boxed region with detailed structure of septum ring post-

expansion by different methods. μMagnify enables visualization of intact septum structure while the other two methods present cracking at the septum rings (Chen2021 and Korovesi2022) and failed expansion (Chen2021). Scatter plot of the root mean square (RMS) error ratio per measurement length (μ) for each ROI by μMagnify (n=6), Chen2021 (n=5) and Korovesi2022 (n=7). Bars for each group represent the mean of error ratio with error bars showing the standard deviation. ANOVA test indicates a significant difference $p < 0.05$ among the three groups. Tukey's HSD test show pairwise difference of distortion between: μMagnify and Korovesi2022 (asterisk, $p<0.05$). **(b)**Yeast form *C. albicans* was fixed with 4% PFA and stained with Dil (green and magenta for pre- and post-expansion images). Samples were subjected to different expansion methods: μMagnify and Chen2021. Deformation field between pre-expansion images (green) taken by Nikon CFI Plan Apo VC 60× C WI (1.2 NA) and post-expansion images (magenta) imaged with the same microscopy setting were overlayed on the registered image in the right column. Biological scales 2 μm. Scatter plot of the RMS error ratio per measurement length for each ROI by μMagnify (n=5), Chen2021 (n=5). Bars for each group represent the mean of error ratio with error bar showing the standard deviation. ANOVA test indicates a significant difference $p < 0.05$ among the three groups. Tukey's HSD test show pairwise difference of distortion between: μMagnify and Chen2021 (asterisk, $p<0.05$). **(c)** *Staphylococcus aureus* (*S. aureus*)-infected U2OS cells were fixed with 4% PFA and stained with DAPI (green and magenta for pre- and post- expansion images) and Dil (yellow). Samples were subjected to different expansion methods: μMagnify, Götz2020[3] and Kunz2021[4]. Deformation field between pre-expansion images (green) taken by Nikon CFI Plan Apo VC 60× C WI (1.2 NA) and post-expansion images (magenta) imaged with the same microscopy setting were overlayed on the registered image in the middle column. Biological scales 2 μm. Boxed region in the left column show the zoom-in views of S. Aureus DNA of pre- and post-expansion images. μMagnify enables consistent DNA expansion between pathogen and host cells while the other two methods present heterogeneous expansion pattern between pathogen and host cells (Götz2020 and Kunz2021). Right column shows lipid retention among different methods, as μMagnify reveals pathogen cell membrane by post-expansion lipid stain (Dil) while others failed. Scatter plot of the RMS error ratio for each ROI by μMagnify (n=17), Götz2020 (n=6) and Kunz2021 (n=6). Bars for each group represent the mean of error ratio with error bar showing the standard deviation. ANOVA test indicates a significant difference $p < 0.05$ among the three groups. Tukey's HSD test show significant pairwise difference between all three groups (asterisk, $p<0.05$).

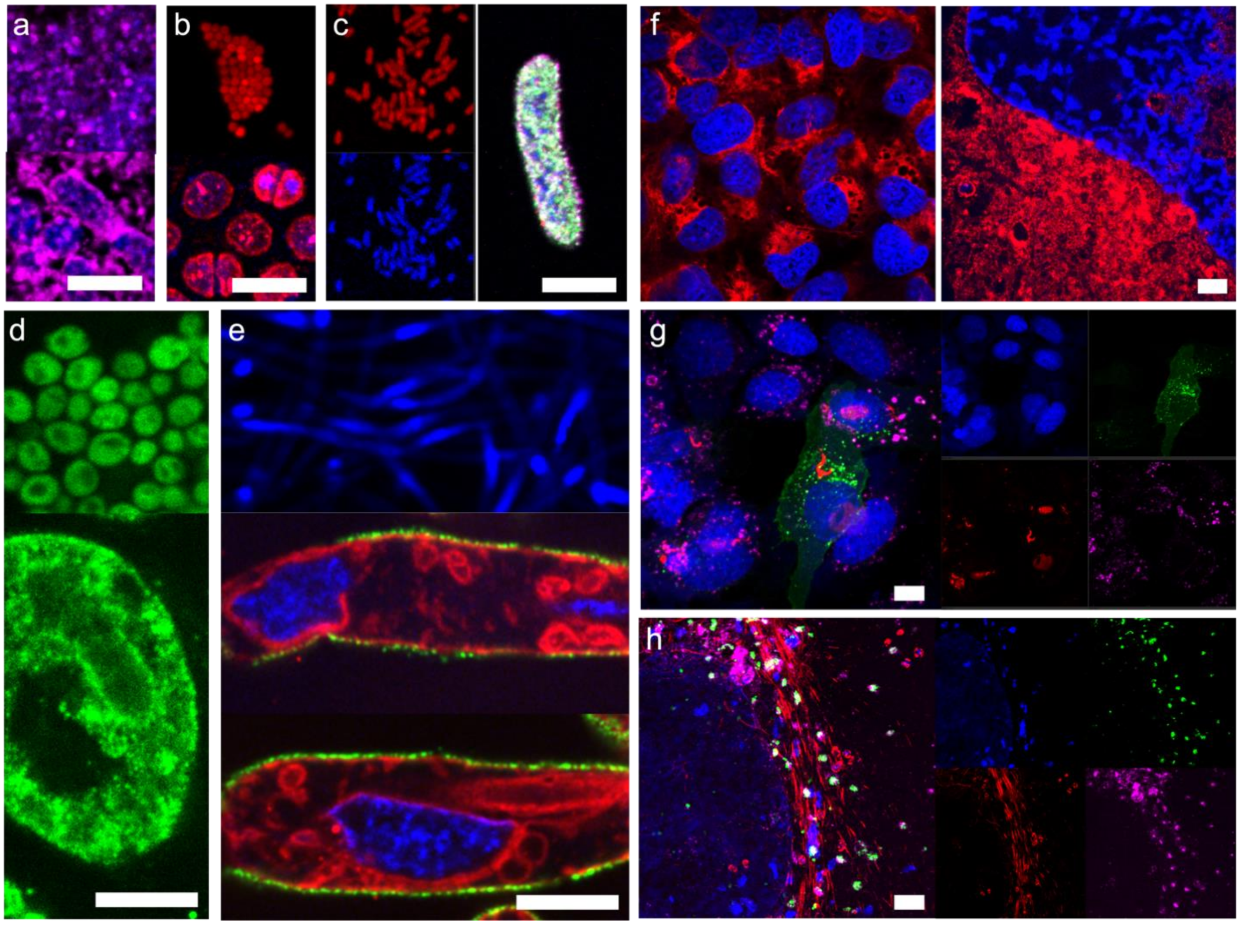

Figure S3: Examples of morphological details revealed by µMagnify comparing to pre-expansion confocal images. Both pre- and post-expansion images were taken at 60x (water). **(a)** Comparison between pre- (up) and post-expansion (bottom) images of *S.P.* D39 strain stained with DAPI (blue) and NHS-AAtto647(magenta). **(b)** Comparison between pre- (up) and post-expansion (bottom) images of *S.A.* that was stained with DAPI (blue, only post-expansion) and Dil (red). **(c)** Comparison between pre- (left) and post-expansion (right) images of *E.C.* that was stained with DAPI (blue) and Dil (red). **(d)** Comparison between pre- (up) and post-expansion (bottom) images of yeast form *C.A.* that was stained with BPDIPY (green). **(e)** Comparison between pre- (up) and post-expansion (bottom) images of *C.A.* hyphae cells that was stained with DAPI (blue), Dil (red, only post-expansion) and LEL (green, only post-expansion). (f) Comparison between pre- (left) and post-expansion (right) images of SA infected U2OS cells that was stained with DAPI (blue), Dil (red). **(g-h)** Comparison between pre- (g) and post-expansion (h) images of U2OS (LITAF-GFP) cells that was stained with DAPI (blue), anti-GFP (green), anti-NEDD4 (red), and anti-CD63 (magenta). On the left is the overlay image with four markers. On the right is montage image of four markers with half-size. All images were presented in physical scales: 10µm.

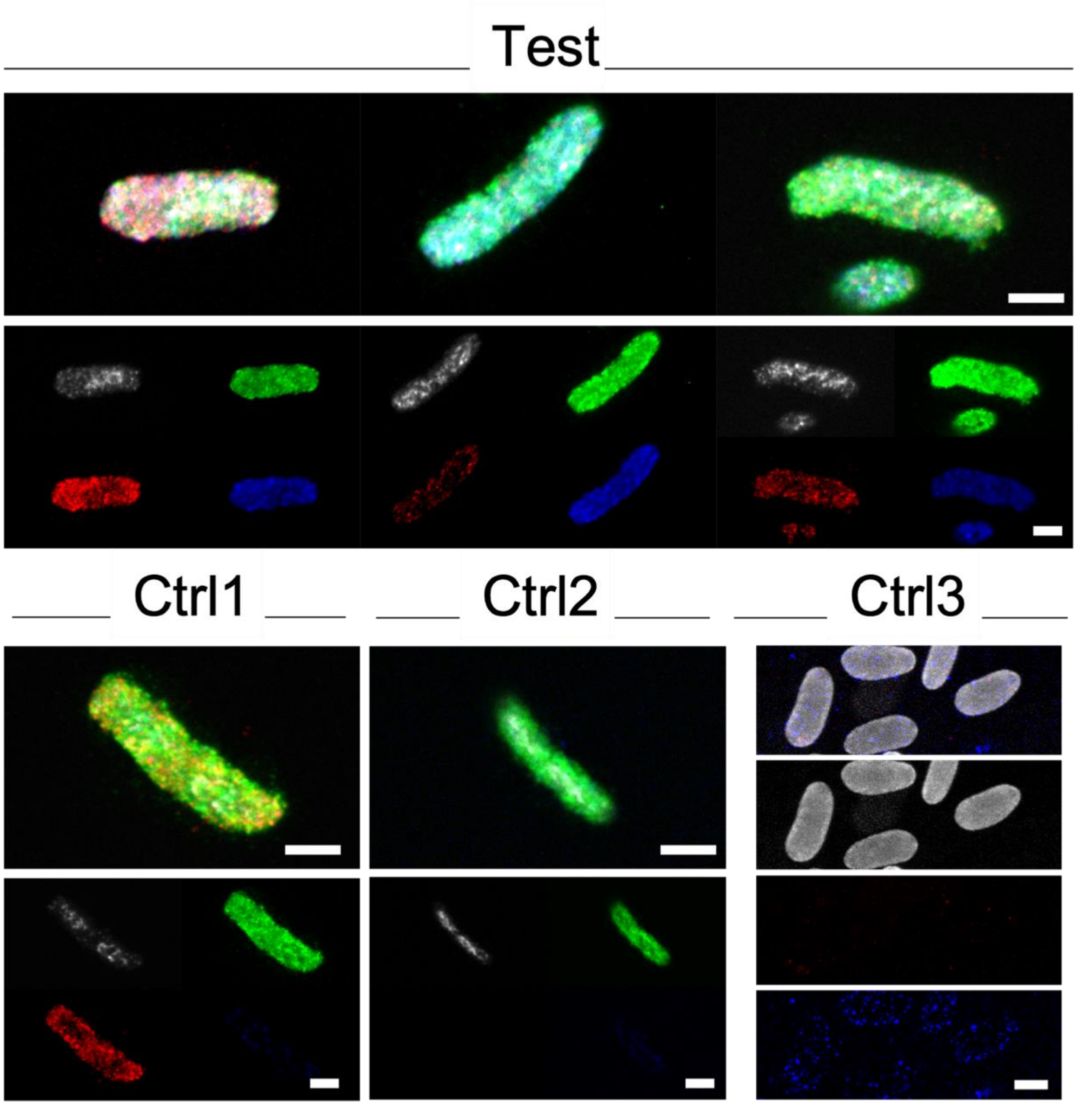

Figure S4: RNA *in situ* hybridization in 4% PFA fixed mNeon expressing E. coli. Test group shows images of *E. coli* with different expression levels of mNeon proteins (green), mNeon RNAs (red) and 16s rRNA (blue). Ctrl1 shows the same sample that stained with mNeon rRNAs (red) and PEG-10 RNA probes (blue). Ctrl2 shows the same sample that stained with only imager probes (the one for mNeon RNA, in red) and mScarlet RNA probes (blue). Ctrl3 shows an uninduced *E. coli*, that carrys an mNeon expressing plasmid. Sample was stained with mNeon RNAs (red) and 16s rRNA (blue). Scale bar: 5 µm.

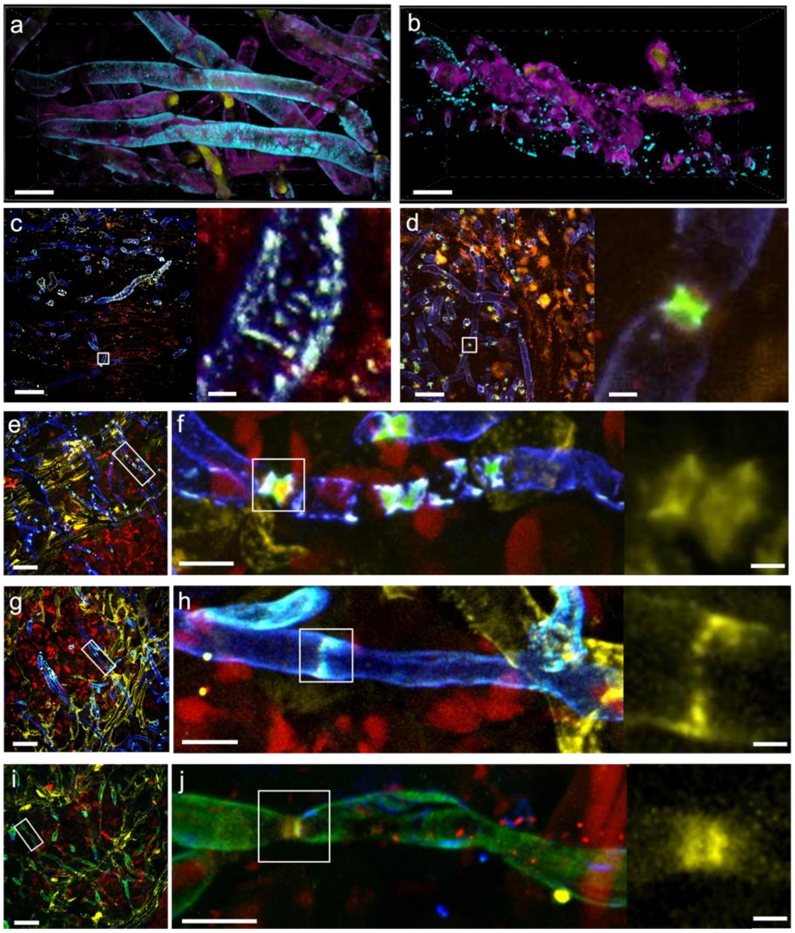

Figure S5. Homogenization optimization. (a-b) Example of under-homogenized and over-homogenized CA biofilm that fixed with 4% PFA. The biofilm was stained with DAPI (yellow), LEL (cyan) and DiI (magenta). **(a)** The gelled biofilm was treated with 20U zymolyase at 37℃ for 48 hr. **(b)** The gelled biofilm was treated with heat denaturation buffer for 24hrs followed by 20U zymolyase digestion at 37℃ for 60 hrs. Scales: 10μm. **(c-k)** Exploration of homogenization condition for H&E stained CA-infected tissue. All samples were stained with DAPI (Red), WGA (Green), NHS-ester (Yellow), LEL (Blue), post-expansion. **(c)** Left: tissue sample was treated with heat denaturation buffer for 60hrs followed by 20U zymolyase digestion for over 7 days. Right: zoom-in view of boxed region. Scales: 50μm (left), 2μm (right). **(d)** Left: tissue sample was digested with 20U zymolysase for 60hrs and then subjected to 60hrs heat denaturation buffer treatment. Right: zoom-in view of boxed region. Scales: 50μm (left), 2μm (right). **(e)** Tissue sample treated with 20U zymolyase for 60hrs and heat denaturation buffer for 48 hrs. (g) Tissue sample treated with heat denaturation buffer RT for 6 hours and 80℃ for 40hrs followed by 40U zymolysase digestion for 24hrs. **(i)** Tissue sample was treated with proteinase K at 60℃ for 3hrs. Scales: 50μm (e,g,i). **(f,h,j)** Zoomed in images of boxed regions in (e,g,i). Scales: 10μm (left), 2μm (right). Characterized expansion factors: 6.6(e), 7.4(h), 5.4(j).

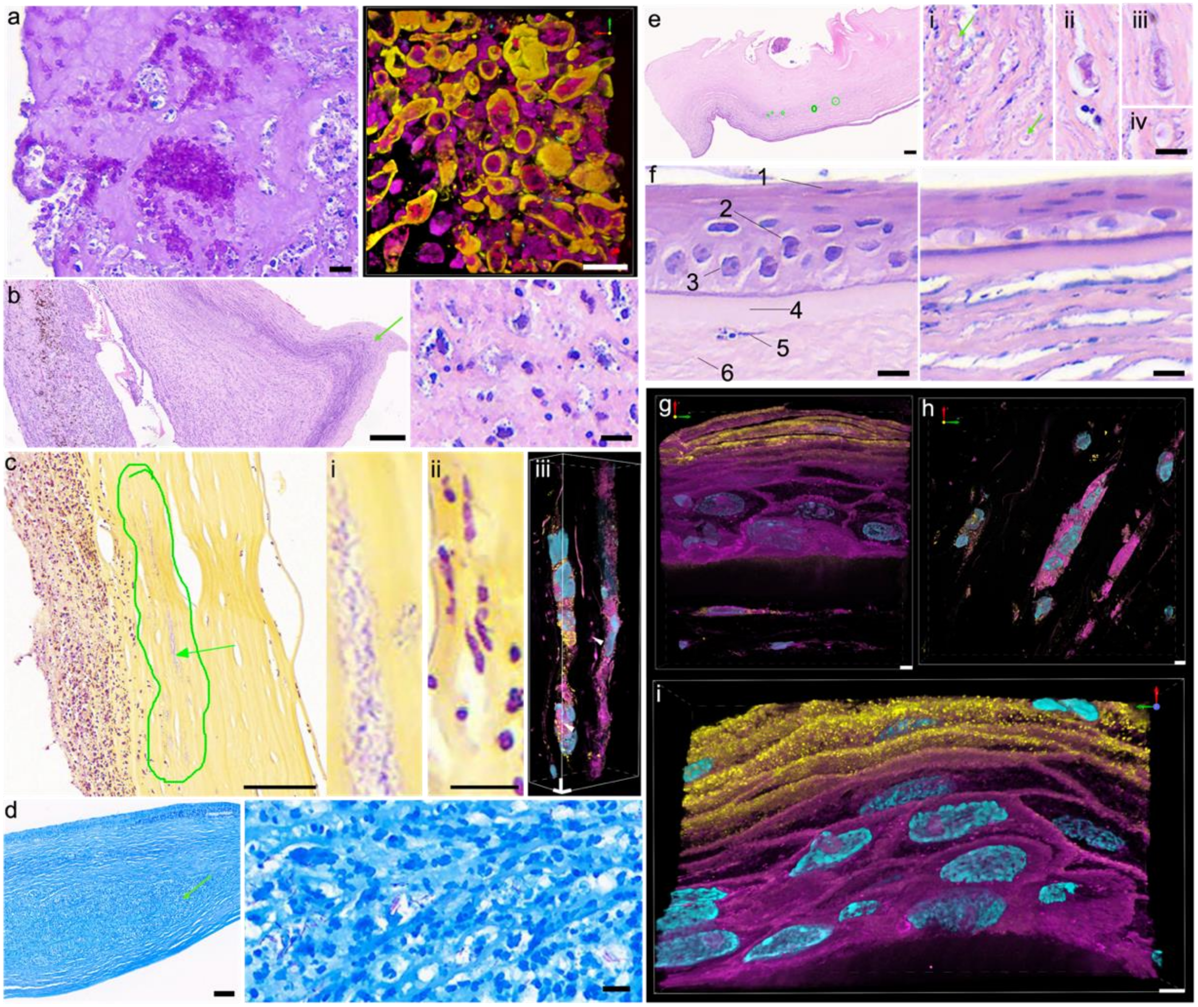

Figure S6: Representative bright field images of different types of microbial keratitis in comparison with µMagnify generated fluorescence images. (a) LEFT: PAS image of *Candida* keratitis of cornea taken at 40x. RIGHT: 3D reconstruction of µMagnify image for dense Candidiasis in cormea, with sample stained by DAPI (cyan), WGA (yellow), Dil (red), and NHS-Atto647N (magenta). Scales: 10µm. **(b)** LEFT: H&E image of eyeball sample with *Staphylococcus epidermidis* keratitis taken at 4x, scale: 250µm. RIGHT: 40x Zoon-in view of green arrow-pointed region on the left. Blue dot reveals the microbes, scale: 10µm. **(c)** Gram stain image of *Pseudomonas* keratitis cornea, scale: 100µm. Representative views of (i) heavily infected spot (outlined and pointed with green arrow), (ii) mild infection of keratocytes that cannot be revealed by bright field image versus (iii) 3D reconstruction of single cell level infection revealed by µMagnify, with sample stained by DAPI (cyan), WGA (yellow), and NHS-Atto647N (magenta). (i-iii) scale: 10µm. **(d)** LEFT: AFB image of cornea tissue with *Atypical mycobacterial* keratitis taken at 4x, scale: 100µm. RIGHT: a zoom-in view of green arrow pointed region on the left. Magenta rod-shape objects reveal the bacteria, scale: 10µm. **(e)** H&E image of *Acanthamoeba* keratitis cornea, with representative views of acanthamoeba infections circled by green outline, scale 100µm. (i) zoom-in view of cell wall (green arrow pointed) of dead acanthamoeba. (ii-iv) zoom-in view of acanthamoeba infection. (i-iv) scale: 10µm. **(f)** Representative H&E images (taken at 40x) of cornea tissue containing: 1. squamous epithelium, 2. polygonal cell, 3. basal epithelium, 4. Bowman's layer, 5. Keratocytes, 6. Collagen fibrils.

Scales: 10 μm.**(g-i)** 3D reconstruction of Confocal fluorescence images of μMagnify processed adjacent normal tissue that was stained with DAPI (cyan), WGA (yellow), NHS-Atto647N (Magenta). (g, h) Epithelium cells. (i) Keratocytes reside in stroma. Scales: 10 μm.

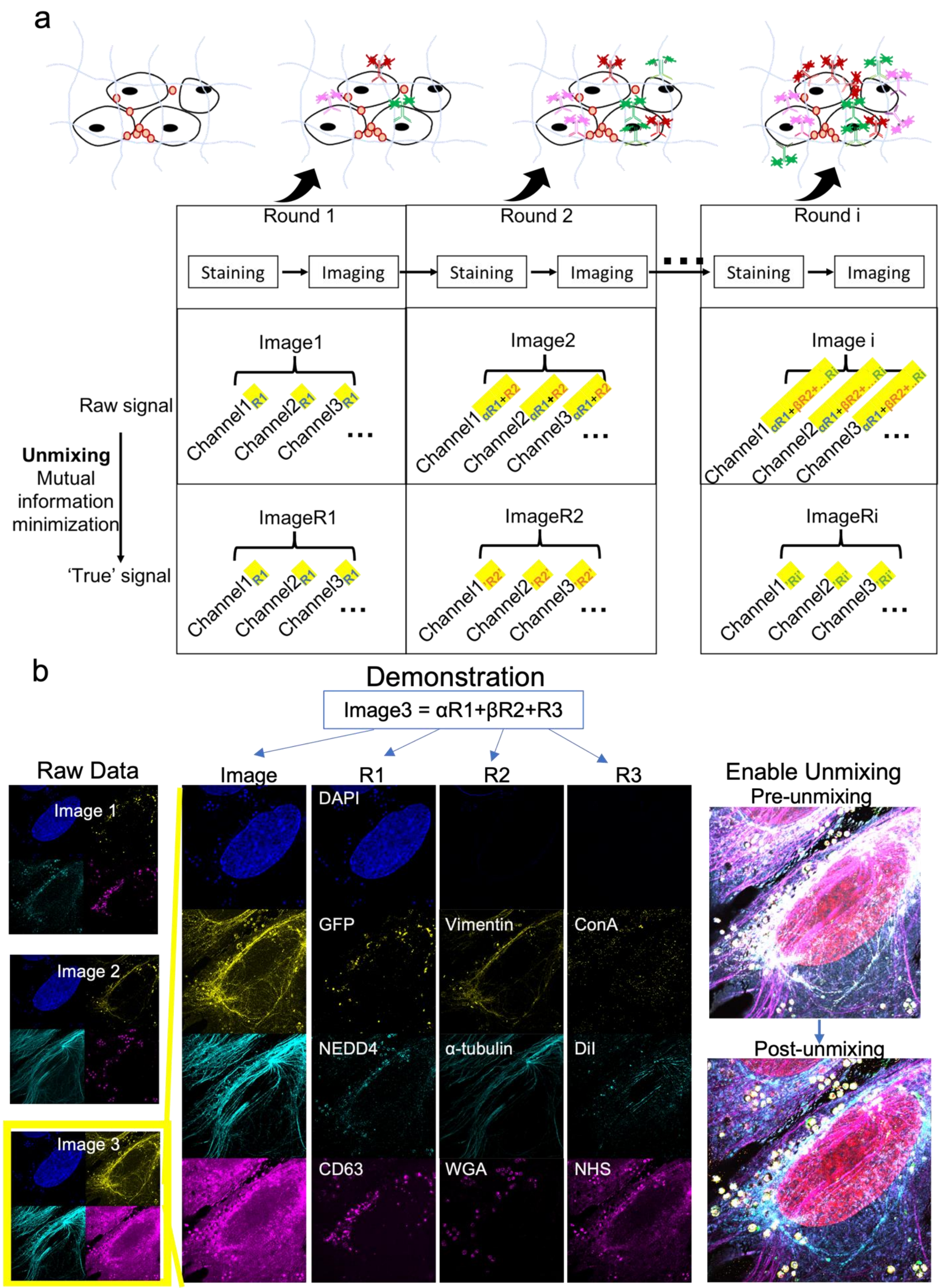
a
Round 1
Round 2
Round i
Staining
Imaging
Image1
Image2
Image i
Raw signal
Channel1
Channel2
Channel3
Unmixing
Mutual information minimization
'True' signal
ImageR1
ImageR2
ImageRi
b
Demonstration
Image3 = αR1+βR2+R3
Raw Data
Image 1
Image 2
Image 3
Image
R1
R2
R3
DAPI
GFP
Vimentin
ConA
NEDD4
α-tubulin
DiI
CD63
WGA
NHS
Enable Unmixing
Pre-unmixing
Post-unmixing

Figure S7: Demonstration of signal unmixing algorithm with three rounds of staining. (a) With all biomolecules of target anchored onto the hydrogel, antibodies and fluorescent labels were accumulatively added to the sample in separate round of staining along with one reference stain. Numbers of stain for each round is determined by the microscope channel capacity. Raw image (image2) captured at the second round consists of the true signal R2 and R1 times a coefficient alpha. Likewise, raw image i captured at round i consists of Ri, R1 times a coefficient alpha, R2 times a coefficient beta, et. al. The true signal of each round (Ri) equals to image i subtracted image i-1 times a coefficient. Enumeration of possible coefficient allows you to resolve an optimal coefficient that minimizes the mutual information between Ri and image i-1. **(b)** For demonstration, S. aureus infected samples were stained for three rounds, with DAPI as reference channel for image registration. Registered raw images of three rounds were shown in the raw image column, with raw signal of each channel is assigned a type of color. Using unmixing algorithm, true signal of each channel of each round could be unmixed, as shown in GFP, vimentin, ConA, NEDD4, alpha-tubulin, Dil, CD63, WGA, and NHS images in the middle column. Pre-unmixing and post-unmixing comparison was shown on the left column with each channel re-assigned an individual color.

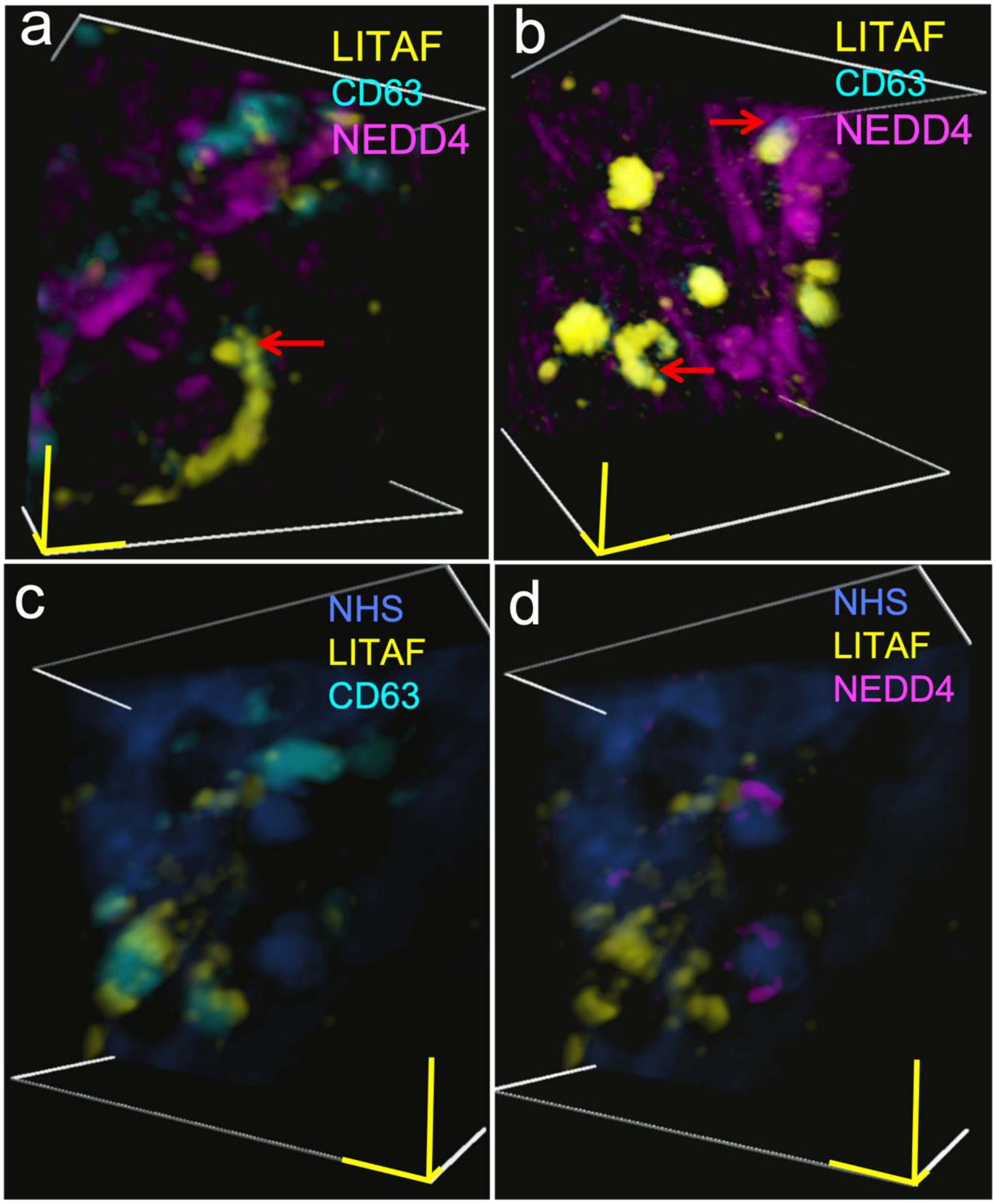

Figure S8. Example images of LIAF&CD63 and LITAFNEDD4 interactions in vacuoles observed by ExMicroVR. Samples (a, mut; b-d wt) were stained with antiGFP (targeting LITAF fusion with GFP), antiCD63, antiNEDD4, NHS Atto647N. Biological scales 1 µm in x, y, z.

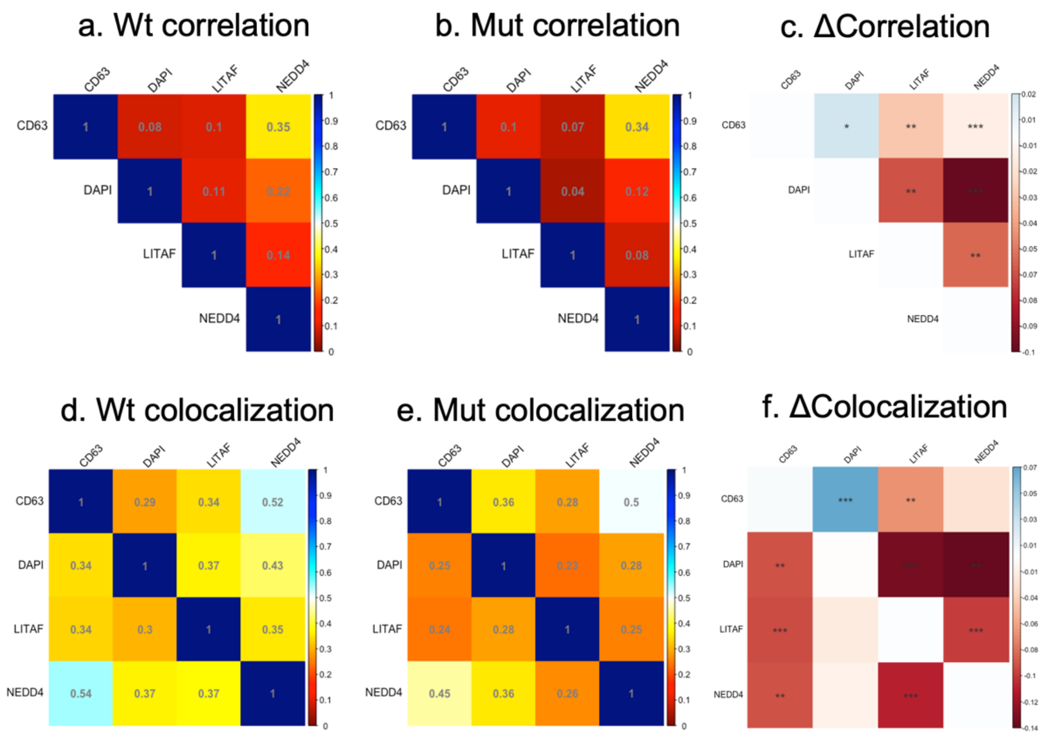

Figure S9: Correlation and colocalization test on pre-expansion confocal images. (a) 4-channel correlation matrix for pre-expansion confocal images capturing SA-infected wildtype U2OS cells (n=13). **(b)** 4-channel correlation matrix for pre-expansion confocal images capturing SA-infected mutant U2OS cells (n=7). **(c)** Delta matrix between matrix in (b) and (a) showing the differences in channel correlations between mutant and wildtype U2OS cells. **(d)** 4-channel colocalization matrix for pre-expansion confocal images capturing SA-infected wildtype U2OS cells (n=13). **(e)** 4-channel colocalization matrix for pre-expansion confocal images capturing SA-infected wildtype U2OS cells (n=7). **(f)** Delta matrix showing the differences in channel colocalization between mutant and wildtype U2OS cells.

Supplementary Tables

Table S1: *Fixations and homogenization and expansion factors for different pathogen samples*

**Sample**	**Fixation**	**Homogenization**		**Expansion factor*** **
		**Denaturation***	**Digestion****	
E. coli suspension	4% PFA	80°C 1hr	37°C 3hrs	7.12±0.40 (N=1, n=45)
S. pneumoniae biofilm	4% PFA	80°C 1hr	37°C 6hrs	7.35±0.46 (N=2, n=83)
C. albicans biofilm	Methanol	80°C 2-8 hrs	37°C 24 hrs	7.23±0.31 (N=2, n=39)
Infected U2OS	4% PFA	80°C 1-3 hrs	37°C 3hrs	7.13±0.09 (N=3, n=16)
Infected mouse tongue	FFPE	RT 8hrs, 80°C 48 hrs	37°C 24 hrs	7.45±0.55 (N=2, n=8)
Infected human cornea	FFPE	RT 8 hrs, 80°C 96 hrs	37°C 24 hrs	3.63±0.32 (N=5, n=5) in PBS<br>8.08±0.55 (N=1, n=4)

** Heat denaturation buffer (1% w/v SDS, 8M Urea, 25 mM EDTA, 2× PBS, pH 7.5 at RT)*
*** Digestion cocktail consists of 500U/mL Mutanolysin, 500U/mL Lysostaphin, 50U/mL Zymolyase, 1kU/mL Collagenase (optional, C2799, Sigma).*
********* *Expansion factors were measured in H2O, except for specially specified. Expansion factor error in terms of s.e.m. over N biological replicates, n technical replicates.*

Table S2: *Polymer synthesis for different pathogen samples*

Monomer solution	Anchoring Methacrolein	Inhibitor stock*	Accelerator stock*	Initiator stock*	Gelling condition	Sample
500 µl	1 µl	1 µl	1.25 µl	10 µl APS	4C 10min, 37 o/n	*E. coli* suspension, *S. pneumoniae* biofilm
500 µl	1.25 µl	1 µl	5 µl	10 µl APS	4C 5-10min, 37 o/n	*E. coli*, *S. aureus*, or *C. albicans* infected U2OS cell culture
500 µl	1.25 µl	1 µl	1.25 µl	10 µl APS	4C 40min, 37 o/n	*C. albicans* infected mouse tongue
500 µl	1.25 µl	4/2 µl	1 µl	50 µl KPS	4C o/n,<br>37 12hrs†	*C. albicans* biofilm, Pathogens infected cornea /eyeball sample

*Initiator stock: 0.5% w/w 4-hydroxy-TEMPO (4HT)
Accelerator stock: 10% w/w TEMED
Initiator stock: freshly made 10% w/w Ammonium Persulfate (APS)
freshly made 5% w/w Potassium Persulfate (KPS)
†Exchange with freshly made gelling solution (inhibitor from 1:125 to 1:250) and allow incubation at 4C 20 min before raise temperature.

Table S3: *RNA FISH probes design*

**Probe ID**	**Target**	**Sequence**
ImagerC2	C2 adaptor	/5Alex546N/CTCTTAGTCAATGCCGCACA
ImagerC4	C4 adaptor	/5ATTO647NN/TGCAATCCTGGCGAACACTC
16srRNA-3C4	16s rRNA	GCTGCCTCCCGTAGGAGTAAGAGTGTTCGCCAGGATTGCA
PEG10-1-3C4	PEG10 mRNA	ttgttgttgttggggggaggTAGAGTGTTCGCCAGGATTGCA
PEG10-2-3C4	PEG10 mRNA	ggtgtgcttggagttgttgtTAGAGTGTTCGCCAGGATTGCA
PEG10-3-3C4	PEG10 mRNA	ggacacacgcactcttatggTAGAGTGTTCGCCAGGATTGCA
PEG10-4-3C4	PEG10 mRNA	cttcttcgttcggtcatgttTAGAGTGTTCGCCAGGATTGCA
PEG10-5-3C4	PEG10 mRNA	ttgatctcttcagagagctcTAGAGTGTTCGCCAGGATTGCA
PEG10-6-3C4	PEG10 mRNA	catgaccttctctcttaagtTAGAGTGTTCGCCAGGATTGCA
PEG10-7-3C4	PEG10 mRNA	ctctgcaggttgttgttctcTAGAGTGTTCGCCAGGATTGCA
PEG10-8-3C4	PEG10 mRNA	tctcgaagggtggtgttctcTAGAGTGTTCGCCAGGATTGCA
PEG10-9-3C4	PEG10 mRNA	tcgatgtcatcatcctcatcTAGAGTGTTCGCCAGGATTGCA
PEG10-10-3C4	PEG10 mRNA	cactcttcctctattggaggTAGAGTGTTCGCCAGGATTGCA
PEG10-11-3C4	PEG10 mRNA	catcgaacttctctgggaggTAGAGTGTTCGCCAGGATTGCA
PEG10-12-3C4	PEG10 mRNA	tgaaaggagccagcatgtctTAGAGTGTTCGCCAGGATTGCA
PEG10-13-3C4	PEG10 mRNA	atgaagatctggcactgggcTAGAGTGTTCGCCAGGATTGCA
PEG10-14-3C4	PEG10 mRNA	gaaatccctggtgctcttttTAGAGTGTTCGCCAGGATTGCA
PEG10-15-3C4	PEG10 mRNA	agacacggacacgatcaactTAGAGTGTTCGCCAGGATTGCA
PEG10-16-3C4	PEG10 mRNA	gtcatcatgcttgtcacgaaTAGAGTGTTCGCCAGGATTGCA
PEG10-17-3C4	PEG10 mRNA	tagttgtgcatcaggtagtgTAGAGTGTTCGCCAGGATTGCA
PEG10-18-3C4	PEG10 mRNA	tgcttcatttccatcatgaaTAGAGTGTTCGCCAGGATTGCA
PEG10-19-3C4	PEG10 mRNA	cctctgagggtcttcaaagaTAGAGTGTTCGCCAGGATTGCA
PEG10-20-3C4	PEG10 mRNA	ctgatcttgcgtttggcaacTAGAGTGTTCGCCAGGATTGCA
PEG10-21-3C4	PEG10 mRNA	ggagtagtcgatgacagaccTAGAGTGTTCGCCAGGATTGCA

PEG10-22-3C4	PEG10 mRNA	tgggcaatcatctggaaagcTAGAGTGTTCGCCAGGATTGCA
PEG10-23-3C4	PEG10 mRNA	tcgtggtactggtcaatcagTAGAGTGTTCGCCAGGATTGCA
PEG10-24-3C4	PEG10 mRNA	tcctgaatgtggtcgctgagTAGAGTGTTCGCCAGGATTGCA
PEG10-25-3C4	PEG10 mRNA	caatcagagcagacagcgacTAGAGTGTTCGCCAGGATTGCA
PEG10-26-3C4	PEG10 mRNA	ccttctctcaatgtgaatgcTAGAGTGTTCGCCAGGATTGCA
PEG10-27-3C4	PEG10 mRNA	cttgcaatgtgaggcaacacTAGAGTGTTCGCCAGGATTGCA
PEG10-28-3C4	PEG10 mRNA	tttctgcgtctttctttttcTAGAGTGTTCGCCAGGATTGCA
PEG10-29-3C4	PEG10 mRNA	acagtagaggcacaggttcaTAGAGTGTTCGCCAGGATTGCA
PEG10-30-3C4	PEG10 mRNA	ggacaattgtcagcgtagtgTAGAGTGTTCGCCAGGATTGCA
PEG10-31-3C4	PEG10 mRNA	cgaagactttgaggccttggTAGAGTGTTCGCCAGGATTGCA
mNeon1_3C2	mNeon mRNA	agagaggccatgttatcctcAATGTGCGGCATTGACTAAGAG
mNeon2_3C2	mNeon mRNA	gtgtaactcatgtgtcgctgAATGTGCGGCATTGACTAAGAG
mNeon3_3C2	mNeon mRNA	caccgttgatggagccaaagAATGTGCGGCATTGACTAAGAG
mNeon4_3C2	mNeon mRNA	tgacccaccatgtcaaagtcAATGTGCGGCATTGACTAAGAG
mNeon5_3C2	mNeon mRNA	aaccatcatttggattgccgAATGTGCGGCATTGACTAAGAG
mNeon6_3C2	mNeon mRNA	gacttcaggtttaactcctcAATGTGCGGCATTGACTAAGAG
mNeon7_3C2	mNeon mRNA	agaactggaggtcacccttgAATGTGCGGCATTGACTAAGAG
mNeon8_3C2	mNeon mRNA	gatatgagggaccagaatccAATGTGCGGCATTGACTAAGAG
mNeon9_3C2	mNeon mRNA	aggtactgatggaagccataAATGTGCGGCATTGACTAAGAG
mNeon10_3C2	mNeon mRNA	gaaaggcgacatcccgtcagAATGTGCGGCATTGACTAAGAG
mNeon11_3C2	mNeon mRNA	ttgtgcgatggacttggtatAATGTGCGGCATTGACTAAGAG
mNeon12_3C2	mNeon mRNA	gaggcaccatcttcaaactgAATGTGCGGCATTGACTAAGAG
mNeon13_3C2	mNeon mRNA	gtgtagcggtagttaacagtAATGTGCGGCATTGACTAAGAG
mNeon14_3C2	mNeon mRNA	ctctcctttgatgtggcttcAATGTGCGGCATTGACTAAGAG
mNeon15_3C2	mNeon mRNA	cgtcagcagggaaaccagtcAATGTGCGGCATTGACTAAGAG
mNeon16_3C2	mNeon mRNA	cagcgagttggtcatcacagAATGTGCGGCATTGACTAAGAG
mNeon17_3C2	mNeon mRNA	ttggggtaagtcttcttcgaAATGTGCGGCATTGACTAAGAG
mNeon18_3C2	mNeon mRNA	ggtactgatgatggttttgtAATGTGCGGCATTGACTAAGAG
mNeon19_3C2	mNeon mRNA	catttccagtggtgtaactcAATGTGCGGCATTGACTAAGAG
mNeon20_3C2	mNeon mRNA	attggcttggcaaaggtgtaAATGTGCGGCATTGACTAAGAG
mNeon21_3C2	mNeon mRNA	gctggttcttcagatagttaAATGTGCGGCATTGACTAAGAG
mNeon22_3C2	mNeon mRNA	cgtcttacggaacacgtacaAATGTGCGGCATTGACTAAGAG
mNeon23_3C2	mNeon mRNA	tcggtcttggagtgcttgagAATGTGCGGCATTGACTAAGAG
mNeon24_3C2	mNeon mRNA	ttgccactccttgaagttgaAATGTGCGGCATTGACTAAGAG
mNeon25_3C2	mNeon mRNA	ccatcacatcggtaaaggccAATGTGCGGCATTGACTAAGAG
mNeon26_3C2	mNeon mRNA	tacttgtacagctcgtccatAATGTGCGGCATTGACTAAGAG
mScarlet1_3C1	mScarlet mRNA	atgaactccttgatcactgcATAATCGCTAGGCACCTGGATT
mScarlet2_3C1	mScarlet mRNA	ctcgatctcgaactcgtggcATAATCGCTAGGCACCTGGATT
mScarlet3_3C1	mScarlet mRNA	acaggatgtcccaggagaagATAATCGCTAGGCACCTGGATT
mScarlet4_3C1	mScarlet mRNA	gagccgtacatgaactgaggATAATCGCTAGGCACCTGGATT
mScarlet5_3C1	mScarlet mRNA	cttatagtagtcggggatgtATAATCGCTAGGCACCTGGATT
mScarlet6_3C1	mScarlet mRNA	cgtcctcgaagttcatcacgATAATCGCTAGGCACCTGGATT
mScarlet7_3C1	mScarlet mRNA	agcttcaccttgtagatcagATAATCGCTAGGCACCTGGATT
mScarlet8_3C1	mScarlet mRNA	cgtcaggagggaagttggtgATAATCGCTAGGCACCTGGATT
mScarlet9_3C1	mScarlet mRNA	ttgtcttcttctgcattacgATAATCGCTAGGCACCTGGATT

mScarlet10_3C1	mScarlet mRNA	atcttaatgtcgcccttcagATAATCGCTAGGCACCTGGATT
mScarlet11_3C1	mScarlet mRNA	cttgtaggtggtcttgaagtATAATCGCTAGGCACCTGGATT
mScarlet12_3C1	mScarlet mRNA	acttgcggtcgacgttgtagATAATCGCTAGGCACCTGGATT
mScarlet13_3C1	mScarlet mRNA	tcgttgtgggaggtgatgtcATAATCGCTAGGCACCTGGATT
mScarlet14_3C1	mScarlet mRNA	tcggagcgttcgtactgttcATAATCGCTAGGCACCTGGATT

Table S4: *P-values of ANOVA test for colocalization matrices between mut and wt*

	Atubulin	CD63	ConA	DAPI	Dil	LITAF	NEDD4	NHS	Vimentin	WGA
**Atubulin**	1.0000	0.0052	0.5282	0.3855	0.4439	0.4154	0.3037	0.4222	0.3343	0.9630
**CD63**	0.0283	1.0000	0.7109	0.6218	0.0925	0.0908	0.0001	0.8359	0.2527	0.5690
**ConA**	0.3288	0.0098	1.0000	0.7989	0.7011	0.4246	0.0341	0.5645	0.2678	0.5398
**DAPI**	0.1693	0.0167	0.5730	1.0000	0.8249	0.3948	0.0006	0.6711	0.9109	0.3124
**Dil**	0.5991	0.0022	0.2967	0.0412	1.0000	0.1540	0.0662	0.0839	0.8659	0.3118
**LITAF**	0.0328	0.0132	0.5412	0.2158	0.4891	1.0000	0.0027	0.3628	0.6733	0.0793
**NEDD4**	0.5052	0.0471	0.3942	0.3385	0.3555	0.5936	1.0000	0.2606	0.4260	0.0700
**NHS**	0.0552	0.0106	0.2553	0.5710	0.1810	0.0625	0.0141	1.0000	0.2660	0.0824
**Vimentin**	0.7740	0.0104	0.1531	0.3051	0.8971	0.9862	0.0491	0.5384	1.0000	0.3856
**WGA**	0.2065	0.2770	0.4416	0.5443	0.5365	0.1135	0.0072	0.4851	0.2624	1.0000

Supplementary Videos

Supplementary Video 1. *ExMicroVR in operation for selective channel rendering and excluder using with sample in figure 5c.*
Supplementary Video 2. *Example of using head-attached excluder to explore thick C.A. biofilm.*

Reference

[1] L. Chen, L. Yao, L. Zhang, Y. Fei, L. Mi, J. Ma, *Front. Phys.* **2021**, *9*, 1.

[2] A. G. Korovesi, L. Morgado, M. Fumasoni, R. Henriques, H. S. Heil, M. Del Rosario, *microPublication Biol.* **2022**, *2022*, DOI 10.17912/micropub.biology.000566.

[3] R. Götz, T. C. Kunz, J. Fink, F. Solger, J. Schlegel, J. Seibel, V. Kozjak-Pavlovic, T. Rudel, M. Sauer, *Nat. Commun.* **2020**, *11*, 1.

[4] T. C. Kunz, M. Rühling, A. Moldovan, K. Paprotka, V. Kozjak-Pavlovic, T. Rudel, M. Fraunholz, *Front. Cell. Infect. Microbiol.* **2021**, *11*, 1.
